# Potential of Steroidal Alkaloids in Cancer: Perspective Insight Into Structure–Activity Relationships

**DOI:** 10.3389/fonc.2021.733369

**Published:** 2021-09-20

**Authors:** Ying Huang, Gen Li, Chong Hong, Xia Zheng, Haiyang Yu, Yan Zhang

**Affiliations:** ^1^Key Laboratory of Computational Chemistry-Based Natural Antitumor Drug Research & Development, Liaoning Province, School of Traditional Chinese Materia Medica, Shenyang Pharmaceutical University, Shenyang, China; ^2^School of Chinese Materia Medica, Tianjin University of Traditional Chinese Medicine, Tianjin, China; ^3^The Second Affiliated Hospital of Liaoning University of Traditional Chinese Medicine, Shenyang, China; ^4^State Key Laboratory of Component-Based Chinese Medicine, Tianjin University of Traditional Chinese Medicine, Tianjin, China

**Keywords:** steroidal alkaloids, structure–activity relationships, anticancer, drug design, medicinal potential

## Abstract

Steroidal alkaloids contain both steroidal and alkaloid properties in terms of chemical properties and pharmacological activities. Due to outstanding biological activities such as alkaloids and similar pharmacological effects to other steroids, steroidal alkaloids have received special attention in anticancer activity recently. Substituted groups in chemical structure play markedly important roles in biological activities. Therefore, the effective way to obtain lead compounds quickly is structural modification, which is guided by structure–activity relationships (SARs). This review presents the SAR of steroidal alkaloids and anticancer, including pregnane alkaloids, cyclopregnane alkaloids, cholestane alkaloids, C-nor-D-homosteroidal alkaloids, and bis-steroidal pyrazine. A summary of SAR can powerfully help to design and synthesize more lead compounds.

## Introduction

**Figure 1:**
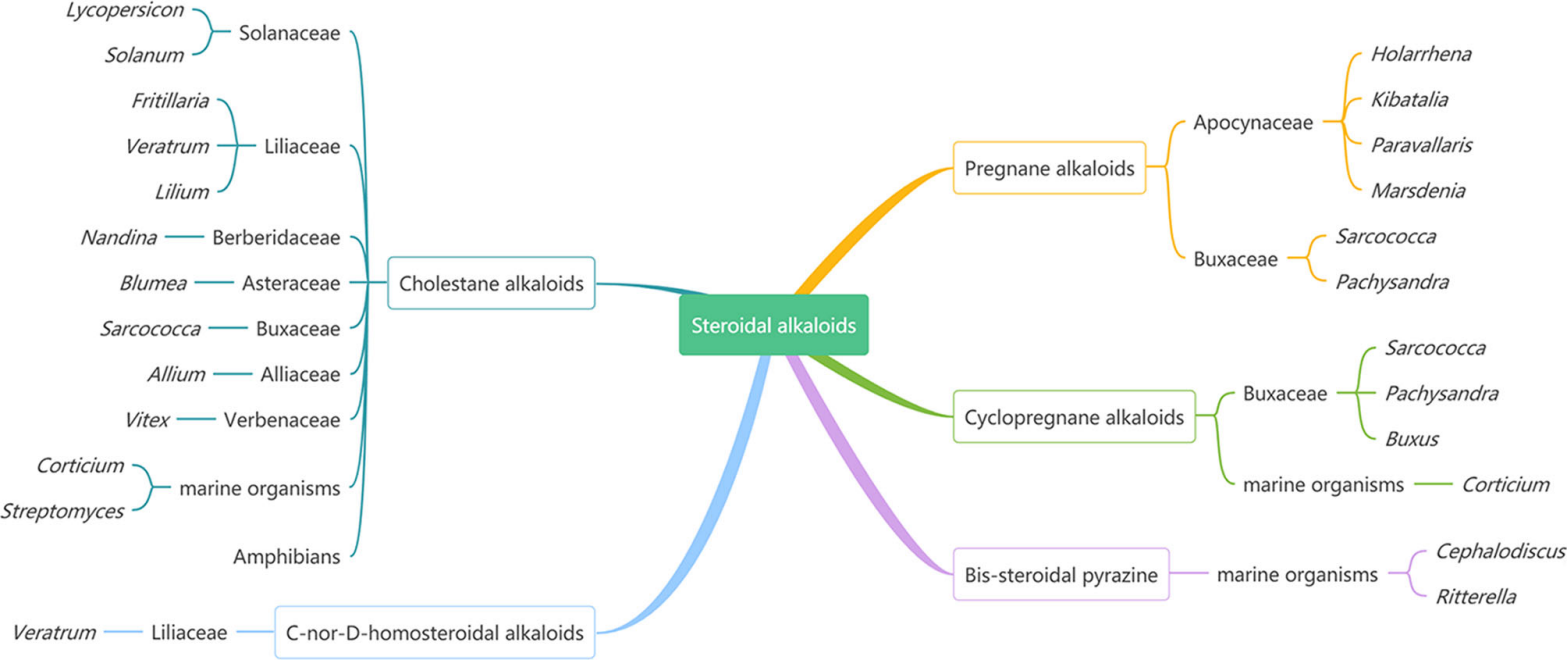
The Main Sources of Steroidal Alkaloids.

Steroidal alkaloids as one large and important class of alkaloids are mainly found in various plants from Solanaceae, Buxaceae, Apocynaceae, and Liliaceae, marine invertebrates, and amphibians (**Figure 1**) (1–3). According to the carbon skeleton, they can be divided into five types: pregnane alkaloids, cyclopregnane alkaloids, cholestane alkaloids, C-nor-D-homosteroidal alkaloids, and bis-steroidal pyrazine (2).

Steroidal alkaloids are nitrogen-containing derivatives of natural steroids, having the basic steroidal skeleton and a nitrogen atom, which contain both steroidal and alkaloid properties in terms of chemical properties and pharmacological activities (4). Therefore, they have significantly outstanding biological activities as alkaloids and similar pharmacological effects to other steroids (4). Previous pharmacological studies have shown that steroidal alkaloids own a variety of pharmacological activities, such as anticancer, anti-inflammatory, antimicrobial, and analgesic (1, 5).

Cancer is considered as one of the most threatening diseases worldwide (6). Currently, the treatments used to treat cancer include four main strategies: chemotherapy, radiotherapy, surgery, and immunotherapy (7, 8). The anticancer natural products include alkaloids (vinblastine, camptothecin), terpenoids (farnesol, geraniol, paclitaxel), anthranilic acid derivatives (tranilast), polyphenolic compounds (gossypol), lignans (podophyllotoxin), and so on (9). Among these, alkaloids and their analogues account for almost the majority of clinical anticancer drugs (10).

Due to the particularity of the structure and potent activities, steroidal alkaloids have received more and more attention for the treatment of cancer in recent years. There are many drugs developed into clinical treatment drugs. At present, most discoveries of new drugs are based on structural modifications through structure–activity relationships. For instance, the hydrochloride of solanamine is used as an antineoplastic drug for preclinical study. Cyclopamine has completed phase I clinical trial as a potential antitumor drug by companies Curis and Genentech (11).

Steroidal alkaloids are important and potential bioactive agents, having a promising feature in the treatment of cancer. More exploitation of new drugs is based on structural modification referring to SAR. Therefore, it is necessary to systematically summarize the structure–activity relationships of steroid alkaloids to design and synthesize potent anticancer drugs.

In this review, any search (including literature and PhD and MSc dissertations) published before 1950 and up to 2020 was performed in the following databases: Web of Science, Science Direct, Scifinder, and the China National Knowledge Infrastructure (CNKI). “Steroidal alkaloids” and “anticancer” were used as keywords in literature searches, and 881 references were obtained in total, of which 189 references meet the requirements. Through these literatures, we summarized the structure–activity relationships of steroid alkaloids in anticancer.

This review focuses on the extensive structure–activity relationships of steroidal alkaloids in the area of anticancer activity. From different carbon skeleton points of view, the differences of activity caused by structural changes were discussed. This review will provide a reference for the discovery of steroidal alkaloid as anticancer drugs.

## Pregnane Alkaloids

**Figure 2:**
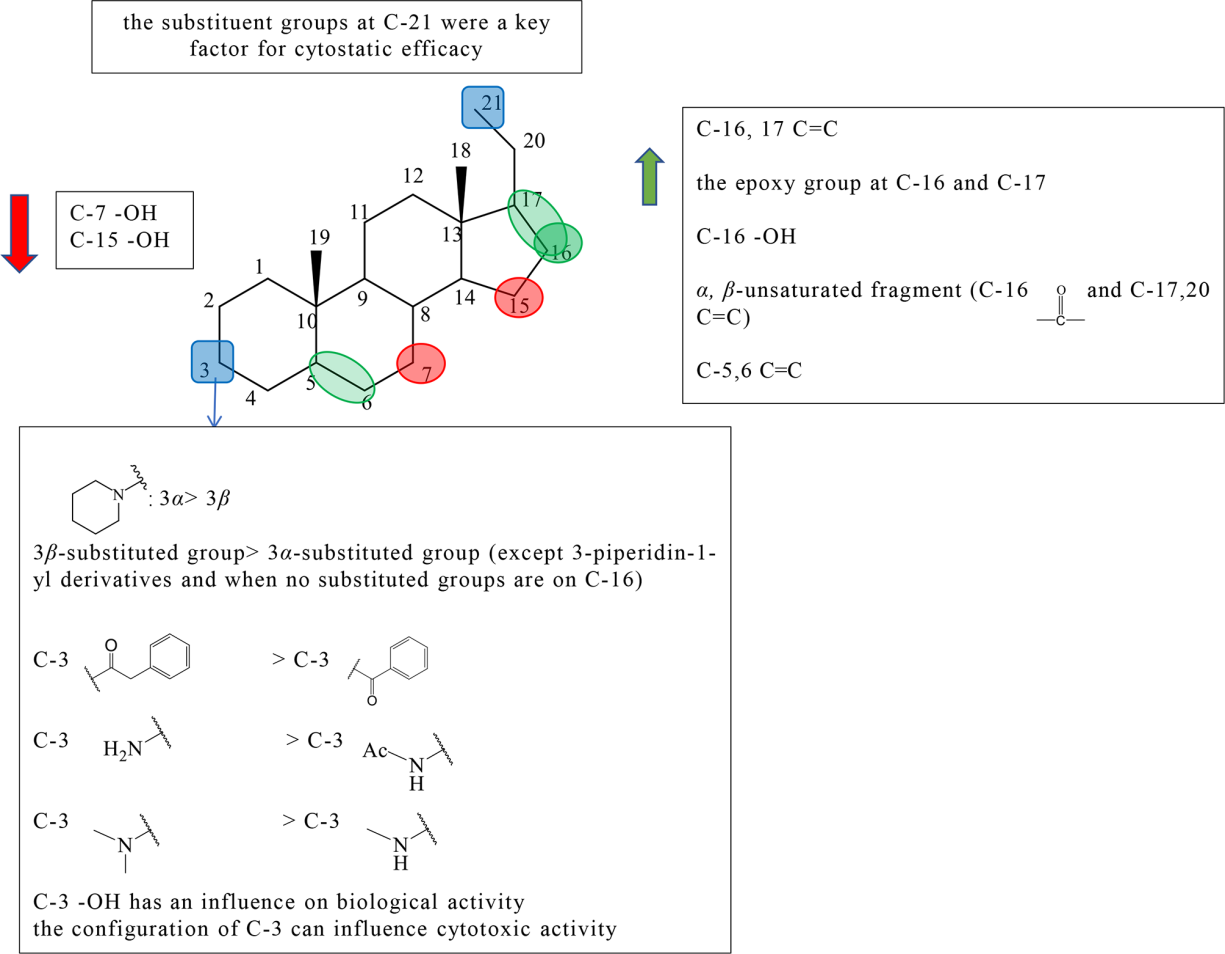
Summary of Structure-activity Relationship of Pregnane Alkaloids.

Pregnane alkaloids are also called C-21 steroidal alkaloids, which are mainly found in the *Holarrhena, Kibatalia, Paravallaris*, and *Marsdenia* genera of Apocynaceae as well as the *Sarcococca* and *Pachysandra* genera of the Buxaceae. Due to their good anticancer activity, pregnane alkaloids have been reported in many literatures, patents, and graduation papers. Based on previous studies, preliminary SAR about the cytotoxic activity of pregnane alkaloids was affected by substituents at C-3, 5, 6, 7, 15, 16, 17, and 21. The schematic diagram of structure–activity relationships of pregnane alkaloids is shown in **Figure 2**.

The substituted forms of C-16 and 17 in pregnane alkaloids are of great importance for the anticancer activity. Huo et al. isolated hookerianine A (1), hookerianine B (2), sarcorucinine G (3), and epipachysamine D (4) from *Sarcococca hookeriana* Baill and evaluated the above three compounds in SW480, SMMC-7721, PC3, MCF-7, and K562 cell lines. Compared to epipachysamine D (4), sarcorucinine G (3) with C-16, C-17 double bond presented better cytotoxicity (**Figure S1**). And hookerianine B (2) with the epoxy group at C-16 and C-17 was stronger than epipachysamine D (4) on cytotoxicity (12). Qin et al. synthesized novel pregn-17(20)-en-3-amine derivatives and performed derivatives of chemotaxis assay against human breast cancer cells (**Table S1**). Although the results demonstrated that the derivatives had no cytotoxicity on MDA-MB-231 cells, some of them displayed strong inhibitory effects against migration such as 5a, 5c, 8e, 8f, 9a, 9g, 10a, and 10f (**Figure S1**). Among these, 10f (IC_50_ value = 0.03 *μ*M) showed the best potency. A majority of C-16 hydroxyl derivatives (7a, 7c-e, 8b, and 8g) presented strong toxicity. A minority of C-16 carbonyl derivatives (10b-c and 9e) presented the toxicity. The derivatives without the substituted group at the C-16 position showed no toxicity, except 5a and 5c (IC_50_ value < 1 *μ*M). The activities of these C-16 carbonyl derivatives including 9a, 9g, 10a, and 10f were better than other two kinds of derivatives, which indicated that *α, β*-unsaturated fragment in ring D might be key for activities. It was worth noting that, except for 3-piperidin-1-yl derivatives, the activities of 3*β*-substituted derivatives were better than 3*α*-substituted derivatives among the derivatives without the substituted group on C-16 (5a-e, 5g *vs* 6a-e, 6g), but it did not appear the same phenomenon among C-16 carbonyl derivatives (13).

The earlier conclusion has been proved that the presence of a 5,6-double bond played an important role in an antiproliferative effect. Compounds 11–13 (the synthetic analogs of solanidine) researched by Minorics et al. showed the effective inhibitory effects against HL-60 cell lines, which verified the earlier conclusion (14). Badmus et al. isolated holamine (14) and funtumine (15) from *Holarrhena floribunda* and tested for their inhibited proliferation against MCF-7, HeLa, HT-29, and KMST-6 (**Figure S1**). Holamine (14) displayed cytotoxic effects on HL-60 and P-388, while funtumine (15) exhibited no cytotoxicity. Therefore, it was speculated that the presence of 5,6-double bond can strengthen cytotoxicity. However, this SAR showed a little effect on the cytotoxic activity against HT-29, HeLa, MCF-7, and KMST-6 cell lines (15).

C-3 is one of the most important positions of the substituent group to affect the activity; the relative configuration of C-3 is also the influential factor. Pregn-17(20)-en-3-amine derivatives by Qin et al. showed results that for compounds with no substituent at C-16, except for 3-piperidine-1-yl derivatives, the activity of 3*β*-substituted derivatives was better than that of 3*α*-substituted derivatives (5a *vs* 6a, 5b *vs* 6b, 5c *vs* 6c, 5d *vs* 6d, 5e *vs* 6e,and 5g *vs* 6g). Among pregn-17(20)-en-3-amine derivatives, the activities of 3*α*-piperidin-1-yl derivatives were better than those of 3*β*-piperidin1-yl derivatives (5f *vs* 6f, 7f *vs* 8f, and 9f *vs* 10f) (**Figure S1**). According to the results, Qin et al. speculated that the relative configuration of the 3*α*-piperidine-1-group might be beneficial for some tumor migration targets, and the 3*α*-piperidin-1-yl group was an effective functional group for the antimigration activity (13). Shaojie Huo et al. concluded structure–activity relationships by comparing hookerianine A (1) and epipachysamine D (4) that steroidal alkaloids with the phenylacetyl group instead of the benzoyl group at C-3 can enhance the cytotoxicity against SW480, SMMC-7721, PC3, and K562 human cells (**Figure S1**) (12). Paravallarine (16), 7*R*-hydroxyparavallarine (17), gitingensine (18), methylgitingensine (19), and *N*-acetylgitingensine (20) were isolated from *Kibatalia laurifolia* and tested for cytotoxic activity against KB cell lines by Phi et al. The results showed that paravallarine (16) presented cytotoxicity with IC_50_ of 12.8 *μ*M, gitingensine (18) and methylgitingensine (19) displayed weak cytotoxicity with IC_50_ ranged from 21 to 42 *μ*M, and *N*-acetylgitingensine (20) showed no cytotoxicity (IC_50_ > 50 *μ*M) (**Figure S1**). It was observed that the acetylation of C-3 amino reduced the cytotoxic activity against KB cells (16). Since paravallarine (16) was more active than methylgitingensine (19), allowing a conclusion that the configuration of C-3 can influence cytotoxic activity (16). Minorics et al. compared the inhibitory effects of compound 12 having the 3-hydroxy group with compounds 11 and 13 containing the 3-acetate group in HL-60 cells. Compound 12 exhibited more effective inhibition activity than compounds 11 and 13 (**Figure S1**). The results indicated that there was an influence on the biological activity when the C-3 hydroxyl group of ring A is present or absent (14). The cytotoxicity of compounds 21 and 22 were evaluated using the NCI-H187 cell line by Cheenpracha et al. (**Figure S1**). Compound 21 displayed weak cytotoxicity against the NCI-H187 cell line with IC_50_ values of 18.2 *μ*M, whereas compound 22 had no cytotoxicity. Based on the results, the methylamino group at C-3 may be better than the *N,N*-dimethylamino group on cytotoxic activity (17).

There are relatively a few studies on C-7, C-15, and C-21. Through previous studies, the following conclusions can be drawn. Kam et al. investigated the cytotoxicity of holamine (23) and 15*R*-hydroxyholamine (24) in HL-60 cells and P-388 cells. Compound 23 exhibited higher cytotoxicity than compound 24, which showed that the C-15 hydroxyl might slightly reduce the cytotoxic activity (**Figure S1**) (18). In the experiment of Phi et al., paravallarine (16) exhibited cytotoxicity against KB cells exhibiting IC_50_ values of 12.8 *μ*M, whereas, 7*R*-hydroxyparavallarine (17) was not cytotoxic (IC_50_ above 50 *μ*M) (**Figure S1**). It can be inspected that the C-7 hydroxyl group leads to the decrease in cytotoxic activity (16). Minorics et al. concluded that the substituent groups at C-21 were a key factor for cytostatic efficacy when compared with 11, 12, and 13 (14).

## Cyclopregnane Alkaloids

**Figure 3:**
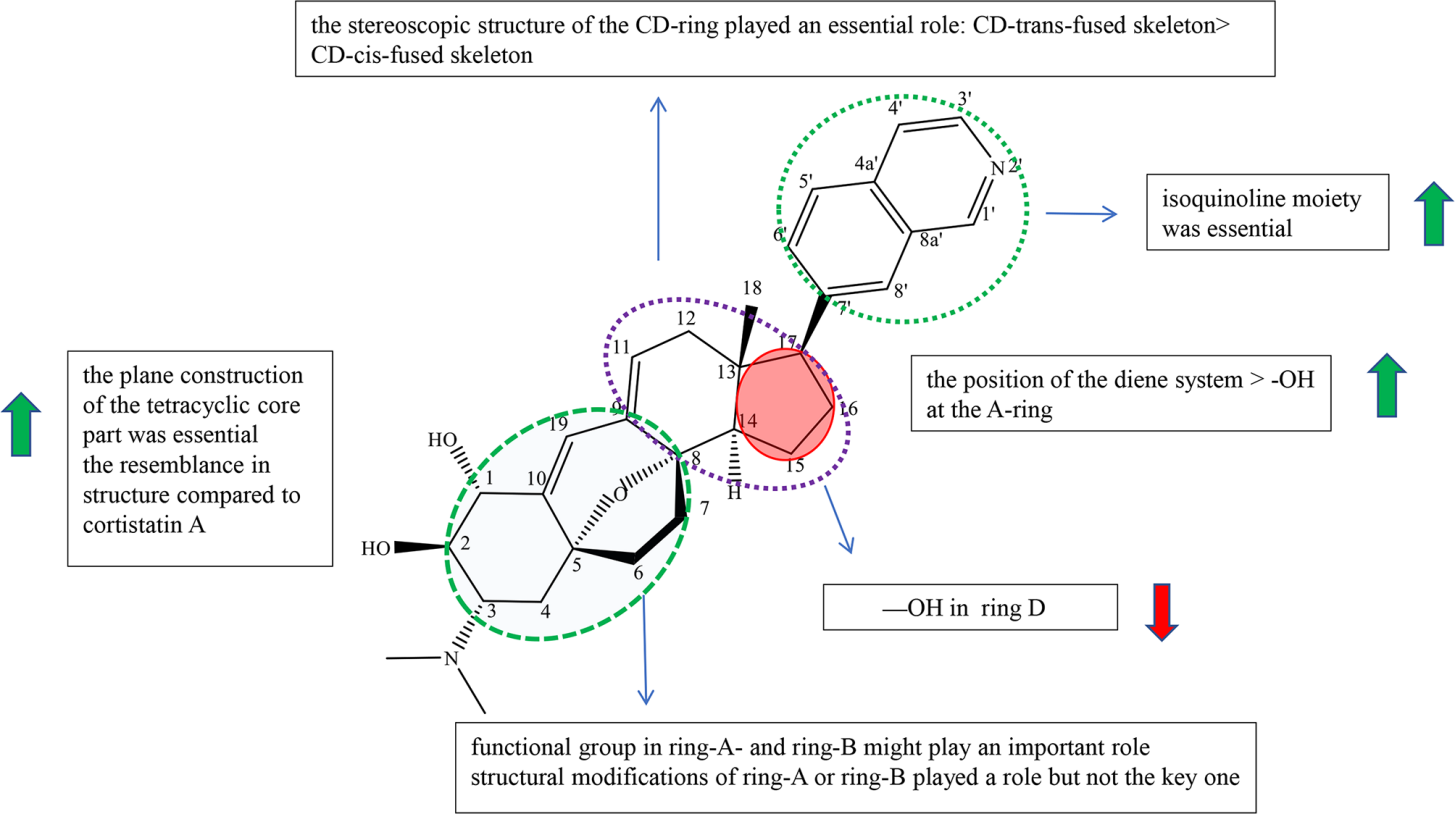
Summary of Structure-activity Relationship of Cyclopregnane Alkaloids.

Cyclopregnane alkaloids are also called C-24 steroidal alkaloids, which contain a characteristic seven-membered system in ring B (1). Among them, cortistatin from marine sponge *Corticium simplex* and cyclopregnane alkaloids from the family Buxus had been widely studied. The schematic diagram of SAR is shown in **Figure 3**.

Cortistatins were first found by Aoki et al., which are compounds with unique structure and potent biological activities, and were isolated from the marine sponge *Corticium simplex* (19). In the reports, Aoki et al. summarized the structure–activity relationships of cortistatin (25-35) (**Figure S2**). Cortistatin E−H (29-32) exhibited weak growth inhibitory activity and poor selective indices against HUVECs, which showed that isoquinoline moiety was essential for potency and selectivity (19). Cortistatin J (33) had more potential with high selectivity than cortistatin K (34) and cortistatin L (35), which suggested that the position of the diene system was of more importance for the activities against HUVECs than the role of the hydroxyl group at the A-ring (**Figure S2**). And it was speculated that the functional group in ring-A and ring-B might play an important role in selective and antiproliferative activity against HUVECs (19). However, cortistatins J (33), K (34), and L (35) showed comparable antiproliferative and selective activities against KB3-1, Neuro2A, K562, and NHDF compared with 25, which indicated that structural modifications of ring-A or ring-B played a role but not a key one (19). Therefore, it is necessary to prove the role of functional groups on ring A in antiproliferative activity through further studies (19).

In 2013, Kotoku et al. converted ring-CD of vitamin D_2_ to analogue 36 of cortistatin A (25), obtained analogue **37** (composition of 37a (8,14-cis) and 37b (8,14-trans)) with a CD-cis-fused skeleton accidentally, and evaluated for cortistatin A (25) and their analogues (**Figure S3**). Cortistatin A (25) and analogue 36 with the CD-trans-fused skeleton exerted antiproliferative potency against HUVEC with IC_50_ at 0.0018 and 0.035 *μ*M, respectively. On the other hand, analogue **37** with the CD-cis-fused skeleton only exhibited weak antiproliferative effect (IC_50_: 1.5 *μ*M) with weak selectivity (ninefold). Furthermore, structures at the CD-ring part of 37a and 37bwere bent, which did not resemble with compound 25 and compound 36 according to molecular mechanics (MM). The results indicated that the stereoscopic structure of the CD-ring of cortistatin A (25) played an essential role in the HUVEC-selective antiproliferative activity (20). Kotoku et al., Aoki et al., and Kobayashi et al. concluded structure–activity relationships through the structure and inhibitory activity of cortistatins B (26) and D (28): the hydroxyl group in ring D led to the decrease in inhibitory activity against HUVECs (**Figure S3**) (19, 21, 22).

The design and synthesis of analogues 38, 39, 40, and 36 of cortistatin A (25) were carried out by Kotoku et al. to improve potent inhibiting activities and antitumor activities (**Figure S3**). Analogues 38 and 40 exhibited moderate growth inhibitory activity against HUVECs (IC_50_: 2.0 and 15 *μ*M) over KB3-1 cells (IC_50_: 18 and 20 *μ*M), whereas analogues 39 and 36 exhibited potential growth inhibitory activity against HUVEC (IC_50_: 0.1 and 0.035 *μ*M) over KB3-1 cells (IC_50_: 10.5 *μ*M each) (21). Moreover, analogues 39 and 36 (>100-fold) showed more selectivity than analogues 38 (9-fold) and 40 (1.5-fold). Besides, the geometrical isomers of 38 and 40 exhibited weaker antiproliferative and selective activity. The results revealed that the plane construction of the tetracyclic core part was essential for selective and antiproliferative activity against HUVECs. The activity of compound 39 was more potential than that of byproduct 41, showing that the resemblance in structure compared to cortistatin A (25) is also an important element (**Figure S3**) (21).

Cyclopregnane alkaloids were mainly found in the genus *Buxus*, containing the basic steroidal skeleton with a cyclopropyl ring at C-9 and C-10. For this kind of compounds, there were a few reports on pharmacological activities, and there are no clear structure–activity relationships for the time being. But according to Qiu’s experiment, we can observe that cyclopregnane alkaloids display strong cytotoxic activity against HeG2 and K562 cell lines through compounds 42, 43, 44, 45, and 46 (IC_50_<6 *μ*M) and showed no cytotoxicity against A549, SW480, SMMC-7721, HL-60, and MCF-7 cell lines through compounds 47, 48, 49, and 50 (IC_50_>40 *μ*M) (**Figure S4**) (23).

On the other hand, compounds 51 and 52 isolated from the genus *Buxus* having a similar structure of cyclopregnane alkaloids showed relatively good cytotoxicity against A-549 and SW480 cell lines.

## Cholestane Alkaloid

**Figure 4:**
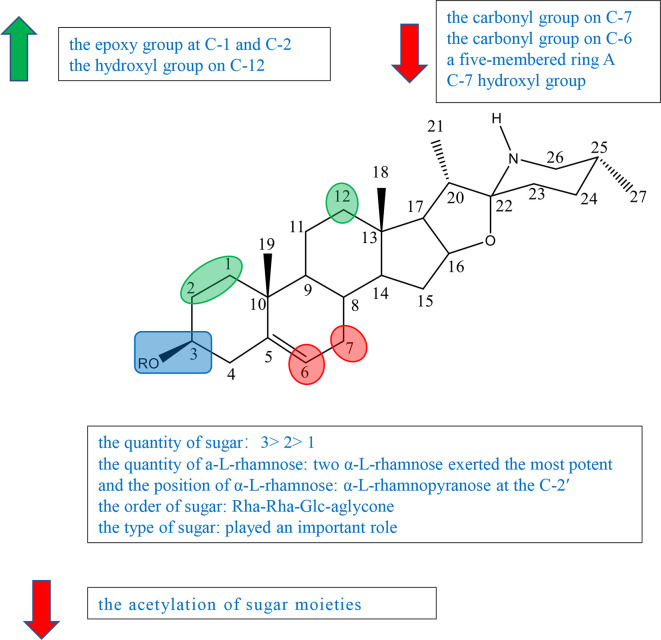
Summary of Structure-activity Relationship of Cholestane Alkaloid—Solasodine (glycoalkaloid).

**Figure 5:**
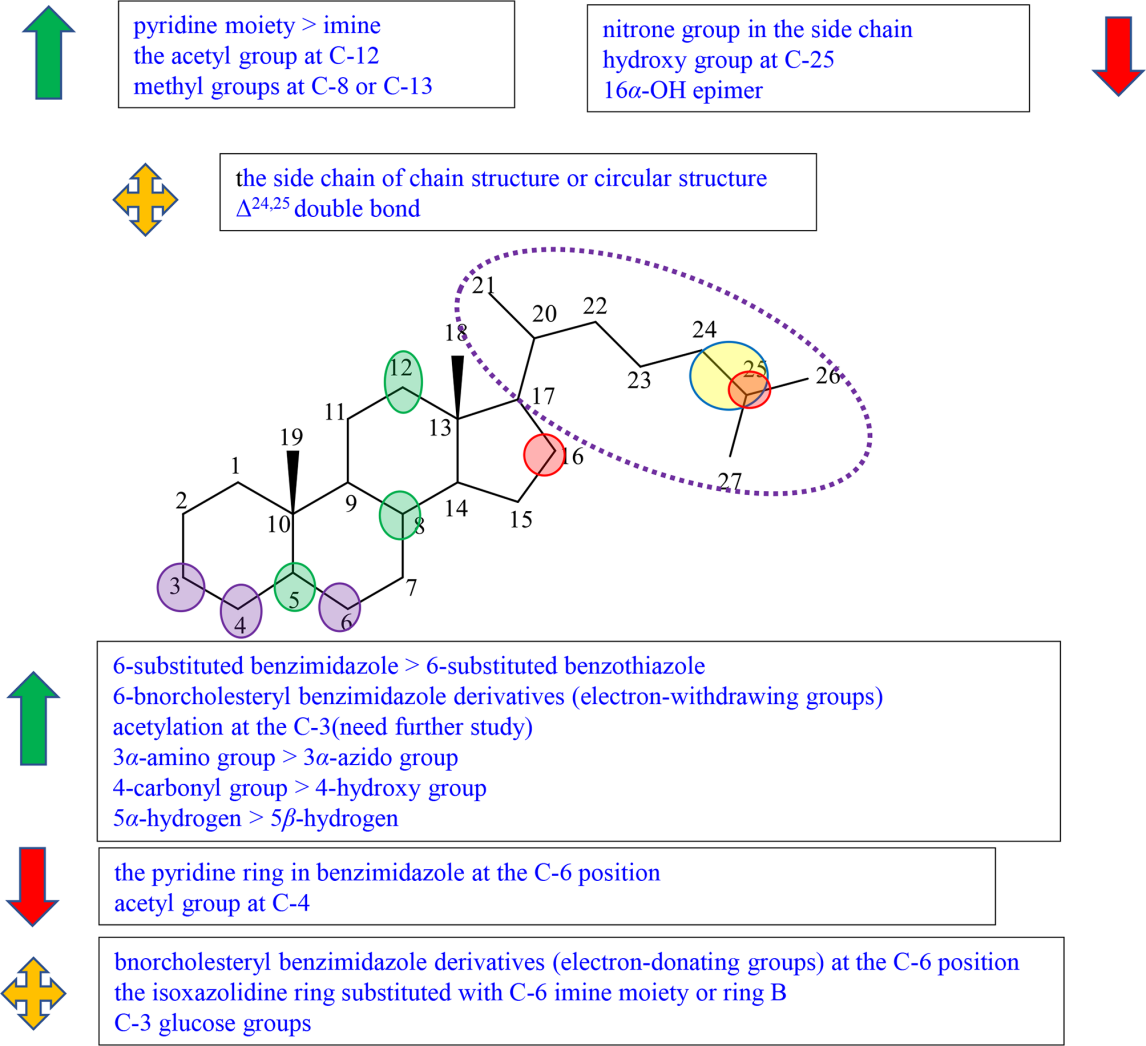
Summary of Structure-activity Relationship of Cholestane Alkaloid (Others).

Cholestane alkaloid is one type of C-27 steroidal alkaloid found in many plants in families Solanaceae and Liliaceae. According to current studies, we divided cholestane alkaloids into solasodine (including glycoalkaloid and not glycoalkaloid) and others to summarize the SAR. The schematic diagram of SAR of solasodine is shown in **Figure 4** and others are shown in **Figure 5**.

### Solasodine (Glycoalkaloid)

Previous studies have indicated that both the aglycone and sugar residues were critical for cytotoxic activity (24, 25). Aglycone was far less inactive than its glycoalkaloid, which showed the essence of sugar moieties (26–28). Some studies also showed that the variation of aglycone did not significantly influence the cytotoxic activity when sugar moieties were kept identical (24, 26, 27). But further study indicated that the activities of some steroidal glycoalkaloid were changed due to the variation of the aglycone. Therefore, it was concluded that the key factors in the activity of solasodine were comprised of two main units, sugar moieties and the substitution of aglycone. The glycosidic moieties included the quantity and the position of *α*-L-rhamnose, and the type and order of sugar (24, 27, 29).

Ding et al. isolated, purified, and identified SS (compound 53), *β*_1_-solasonine (compound 54), SM (compound 55), *β*_2_-solamargine (compound 56), *γ*-solamargine (compound 57), and solanigroside P (compound 58) from *Solanum nigrum* L and investigated their anticancer activity against MGC-803 cells by MTT (**Figure S5**). They noted that steroidal alkaloids containing trisaccharides (compounds 53 and 55) showed better results on activity than those containing disaccharides (compounds 54 and 56) or monosaccharides (compound **57**). Compound 54 containing three sugar units with two *α*-L-rhamnose had the highest antiproliferative activity among compounds 51–56, which suggested that the quantity of *a*-L-rhamnose was critical for the activity (**Figure S5**) (29). In 2015, Akter et al. isolated SGA 1 (compound 59) from the leaves of *Blumea lacera* and tested the cytotoxicity of SGA 1 and SGA analogues including *β*-solamargine (compound 60), *α*-solamargine (compound 61), and khasianine (compound 62) (**Figure S5**). Khasianine exhibited weak cytotoxic effect on MCF-7 cells. SGA 1 and *β*-solamargine displayed remarkable cytotoxicity against all tested cell lines. A comparison of khasianine, SGA, and *β*-solamargine was performed, in which aglycone of glycoalkaloids were the same, but the quantity and the position of *α*-L-rhamnose were varied. The results suggested that the second additional Rha (Rha-Rha-Glc-aglycone) led to the significant enhancement of the activity, and the third additional Rha (Rha-Rha-Glc(Rha)-aglycone) had little further effect on cytotoxicity. Furthermore, it is concluded that two terminal *α*-L-Rha have positive influence on cytotoxicity (24).

Chang’s study indicated that *α*-solamargine (compound **61**) with (*α*-L-Rha-(1-2)-*α*-L-Rha-(1-4)-*β*-D-Glc-(1-aglycone) showed significant activity against human hepatoma cells, but khasianine (compound 62) with (*α*-L-Rha-(1-4)-*β*-D-Glc-(1-aglycone) exhibited weak activation effect. The comparison between *α*-solamargine and khasianine showed that the 2′-rhamnose moiety may impact significantly on triggering cell death. It was suggested that the enhancement of the biological activity was because the dihedral angle of the glycosidic bond is changed by 2′-rhamnose moiety (26). Among the six compounds isolated from *Blumea lacera* by Ding et al., compounds 53, 54, and 55 having *α*-L-rhamnopyranose attached to C-2 of *β*-D-glucose or galactose displayed significant cytotoxic effects against MGC-803 cells. Compound 56 with *a*-L-rhamnopyranose at the C-4 and compound **57** without *a*-L-rhamnopyranose showed little antiproliferative activities. The results inspected that *a*-L-rhamnopyranose at C-2 was important for inhibition activities (29). Compound 54 had far higher activity than compound 56, suggesting that the type of sugar or the position of rhamnose had an effect on the activity (29). Xiang et al. investigated the cytotoxicity of five steroidal glycoalkaloids isolated from *Solanum nigrum* L. against five human cancer cell lines including HL-60, U-937, Jurkat, K562, and HepG2 cell lines. Compound 66 with the same aglycone as 67 had greatly higher activity than 67, showing that the type of sugar moiety played an important role in cytotoxic activity (**Figure S5**) (29).

Esteves-Souza et al. reported the inhibited cell proliferation evaluation of solasonine (compound 68) and its acetylation. Solasonine (compound 68) showed antiproliferative effect against Ehrlich carcinoma cells with IC_50_ at 74.20 ± 6.26 *μ*M, but 68a furnished by the acetylation of 68 was inactive, suggesting that the acetylation of sugar moieties may reduce the antiproliferative activity (**Figure S5**) (27).

Liu et al. investigated the synthesis of 1-*α*-hydroxysolanine (compound 69), obtained a series of intermediate products and unexpected products from diosgenin, and tested their cytotoxicity (**Figure S5**). Among the compounds tested [compounds 70, 71–74, and solasodine (75)], only epoxide 75 had moderate inhibiting activities against PC3, Hela, and HepG2 cells lines at the concentration of 10 *μ*M, suggesting that the epoxy group at C-1 and C-2 might impact the cytotoxic activity (**Figure S5**) (30).

In the experiment of Ding et al., compound 58 having the same sugar moieties as compound 56 was more active (IC_50_ = 20.10 *μ*g/ml) than compound 56. The results suggested that the hydroxyl group on C-12 of steroidal alkaloid skeleton may be a factor on the activities (29).

Among steroidal glycoalkaloids tested by Xiang et al., compounds 63–66 contain identical sugars, but the aglycones are variable in rings A and B. Compound 66 showed the most powerful potency to all cell lines, but compounds 63–65 exhibited no cytotoxicity (**Figure S5**). Based on the result, it was speculated that the carbonyl group on C-6/7 (compounds 64 and 65) and the five-membered ring A (compound 63) led to the decrease in anticancer activity (25).

Gu et al. isolated 7*α*-OH solamargine (compound 76) and 7*α*-OH solasonine (compound 77) together with known compounds 78 and 79 from the fruits of *Solanum nigrum* (**Figure S5**). Compounds 78 and 79 displayed cytotoxic activities against MGC803, HepG2, and SW480 cell lines with the IC_50_ values ranging from 7.02 ± 0.60 to 23.79 ± 1.42*μ*M. On the other hand, compounds 80 and 81 containing hydroxyl groups located at the C-7 showed no cytotoxicity, suggesting that the C-7 hydroxyl group of the aglycone decreased the cytotoxicity (**Figure S5**) (31).

### Solasodine (Not Glycoalkaloid)

In 2012, Zha et al. synthesized novel solasodine derivatives and investigated their cell growth inhibitory effect against PC-3 cell lines. Analogue 91 displayed the highest antiproliferative activity against PC-3 cell line among solasodine derivatives (compounds 82–92), but analogue 90 (3*β*-hydroxyl) and analogue 92 (3*β-p-*tertbutylbenzoyl) with the similar structure as analogue 91 showed inferior activity (**Figure S6**). The results suggested that substituent groups at C-3 may play a role in inhibitory activities *in vitro*. Moreover, solasodine derivatives that performed etherization and esterization at C-3 (analogues 84–89), except analogue 89 (1-naphthoyl), did not show enhancement of the antiproliferative activity (32). In the same year, Zha et al. also reported a series of structural modification of soladulcidine and tested inhibitory effects of synthetic compounds against PC-3 cell lines *in vitro*. Among the C-3 hydroxyl group modified compounds, soladulcidine 93 showed inhibitory effects against prostate gland adenocarcinoma (PC-3) cell line, and its derivatives 94–100 were inactive, suggesting that hydroxyl groups at C-3 were better than other substituents tested (**Figure S6**) (33).

### Others

Sunassee et al. reported the growth inhibition against National Cancer Institute (NCI) 60 cell lines of Plakinamines N (101), O (102) I (103), and J (104), which were isolated from *Corticium niger* by guided separation technology (**Figure S7**). Plakinamines N (101), O (102), and J (104) with a substituted pyrrolidine ring showed potent cytotoxicity against all 60 cell lines with IC_50_ of 11.5, 2.4, and 1.4 *μ*M, while plakinamine I (103) with the fused piperidine ring system displayed only modest activity. It was speculated that the steroidal side chain at C-20 may influence the cytotoxic activity (34). In the experiment performed by Abdel-Kader et al., they tested compounds isolated from *Eclipta alba* cytotoxicity against the M-109 cell line and activity against *Candida albicans* including (20*S*, 25*S*)-22,26-imino-cholesta-5,22(*N*)-dien-3*β*-ol (verazine, 105) ecliptalbine [(20*R)*-20-pyridyl-cholesta-5-ene-3*β*,23-diol] (106), and 25*β*-hydroxyverazine (107) (**Figure S7**). Although they believed that antifungal activity is the main activity of these compounds rather than antitumor activity due to the weak cytotoxicity, it also can be observed the structure–activity relationships of cytotoxicity by Abdel-Kader et al. The cytotoxicity of compound 105 was greater than that of compound 106, which might be due to the change of the imine to a pyridine moiety in the side chain (35). Lee et al. reported the cytotoxicity of lokysterolamine A (108), plakinamine E (109), and plakinamine F (110) isolated from the sponge *Corticium* sp. (**Figure S7**). We can observe that the nitrone group in the side chain slightly reduced cytotoxicity against the human leukemia cell line K562 by comparing plakinamine E (109) with lokysterolamine A (108) (36). Zampella et al. isolated plakinamine I (compound 103) from the marine sponge *Corticium* sp., synthesized their derivatives, compounds 113 and 114, and evaluated for their cytotoxicities against MCF7 cell lines (**Figure S7**). Their results showed that the cytotoxicity of compounds 113 and 114 were relatively good in comparison to plakinamine I (compound 103), suggesting that the side chain of the chain structure or the circular structure exerts no obvious influence on cytotoxicity (37).

Ridley et al. reported the isolation and identification of plakinamine I-K (103, 104, 115) and dihydroplakinamine K (116) from the marine sponge *Corticium niger* (**Figure S7**). These compounds were tested for cytotoxicity against the human colon tumor cell line HCT-116. Plakinamine K (115) and dihydroplakinamine K (116) having the same skeleton apart from the presence of double bond at C-24 and C-25 showed comparable potency against the human colon tumor cell line HCT-116, suggesting that the presence of the Δ^24,25^ double bond had a little or no effect on cytotoxicity (38). Abdel-Kader et al. compared 25*β*-hydroxyverazine (107) with verazine (105), concluding that the introduction of the hydroxy group at C-25 reduced the cytotoxic activity against the M-109 cell line (35).

Fedorov et al. synthesized 14 steroidal alkaloids (117–130) containing benzimidazole or benzothiazole moieties, with the compounds from Japanese sponge *Stelletta hiwa aensis* as the parent cores (**Figure S7**). They tested the cell inhibitory effects of these compounds against HeLa and HepG2 cell lines and summarized the structure–activity relationships. The SAR study showed that the 6-substituted benzimidazole compounds were more active than the 6-substituted benzothiazole compounds regarding inhibitory effects by comparing compound 121 with compound 129 (39). Among bnorcholesteryl and benzimidazole derivatives, electron-withdrawing groups led to the decrease in cytotoxicity such as compound 118, compound 120, and compound 122, and electron-donating groups had little influence such as compound 121, compound 123, and compound 125 (39). In comparison with compounds 119, 127, and 128, the isoxazolidine ring substituted with C-6 imine moiety or ring B did not play a positive role in anticancer activity. In comparison with compounds 121, 127, and 130, the pyridine ring in benzimidazole can reduce the cytotoxic activity (39). It is also at the C-6 position wherein, dendrogenin A (DDA, compound 131) was a potential derivative, which contains imidazol joined with 6-ethylamino founded by Sandrine Silvente-Poirot and co-workers, displaying anticancer properties (40, 41).

Sun et al. isolated 3-*O*-acetylveralkamine (132) and veralkamine (133) from *Veratrum taliense*, and they were evaluated for their cytotoxic effects against HL-60, SMMC-7721, A-549, MCF-7, and SW480 cell lines (**Figure S7**). Compound 132, regarded as the acetylated derivative of compound 133, had higher activity than compound 133, suggesting that acetylation at the C-3 position may enhance the cytotoxic activity (42). This conclusion that compound 121 was more active than compound 122 was also proved in the experiment of Fedorov et al. (**Figure S7**) (39). However, some experimental results did not fit this inference. For example, compounds 118 and 120 with the acetyl group at the C-3 position were less active than compounds 117 and 119 with the hydroxy group at the C-3 position, respectively (**Figure S7**). It was deduced preliminarily that these results may be due to electron-withdrawing groups of the benzimidazole at C-6 (39). Fuchs’ team showed that the cytostatic activity of compounds with the acetylated hydroxy group at C-3 was lower than that of derivatives with the free hydroxy group at C-3 (43). Therefore, it is necessary to have further study in order to verify the correctness of the conclusion. Compounds 111 and 112 and their amination products 111a and 112a, the intermediate products in the process of synthesizing derivatives of 19-acetoxy-3*α*-amino-5*α*-cholestane, were synthesized by Zampella et al. (**Figure S7**). Compounds 111a and 112a possessed no cytotoxic activity, and compounds 111 and 112 exhibited good cytotoxicity. The results revealed that the 3*α*-amino group played an important role in observed cytotoxicity rather than the 3*α*-azido group (37).

In the experiment of Sunassee et al., the comparison between plakinamines O (102) and J (104) indicated that substitution of the acetyl group at C-4 may cause the decrease in cytotoxic activity (**Figure S7**) (34). In the experiment of Lee et al., lokysterolamine A (108) showed superior activity against the human leukemia cell line K562 compared to plakinamine F (110), showing that a 4-hydroxy group may be a better substituent group than the 4-carbonyl group on cytotoxic activity (**Figure S7**) (36).

Among compounds 137–143 (compounds 137–140 with the acetyl group at C-12, compounds 138–140 with the glucose group at C-3) isolated from *Veratrum grandiflorum Loes* and tested for inhibition activities on the Hh pathway by Gao et al., compounds 139–141 and 143 showed prominent inhibitory activity, and compounds 142, 144, and 145 exhibited no inhibitory activities (**Figure S7**). A primary SAR study showed that the acetyl group at C-12 may play an important role on the activity, and the C-3 glucose groups had little influence on the activity (44).

In Zampella’s experiment, compound 137 with 5*α*-hydrogen showed better activities than compound 138 with 5*β*-hydrogen, which revealed that the relative configuration of hydrogen at C-5 may have an effect on cytotoxicity (**Figure S7**) (37). In order to find compounds with antitumor activity in natural plants, Wang et al. studied the antitumor effects of steroidal alkaloids and extracted them from different solvents including chloroform, *n*-hexane, and water from cultivated Bulbus *Fritillariae ussuriensis*. Verticine as well as imperialine were isolated and investigated their cytotoxicity against LLC, A2780, HepG2, and A549 cells lines. It can be observed that the activity of verticine was far higher than that of imperialine against LLC, and slightly higher against HepG2 and A549 cells lines, implying that methyl groups at C-8 or C-13 can enhance the cytotoxicity (45).

Fuchs’ team demonstrated that the configuration of the C-16-OH played an important role in cytotoxic activity. The 16*α*-OH epimer displayed weak effect on cytotoxicity even at 0.1 *μ*M concentration (43).

## C-nor-D-Homosteroidal Alkaloids

**Figure 6:**
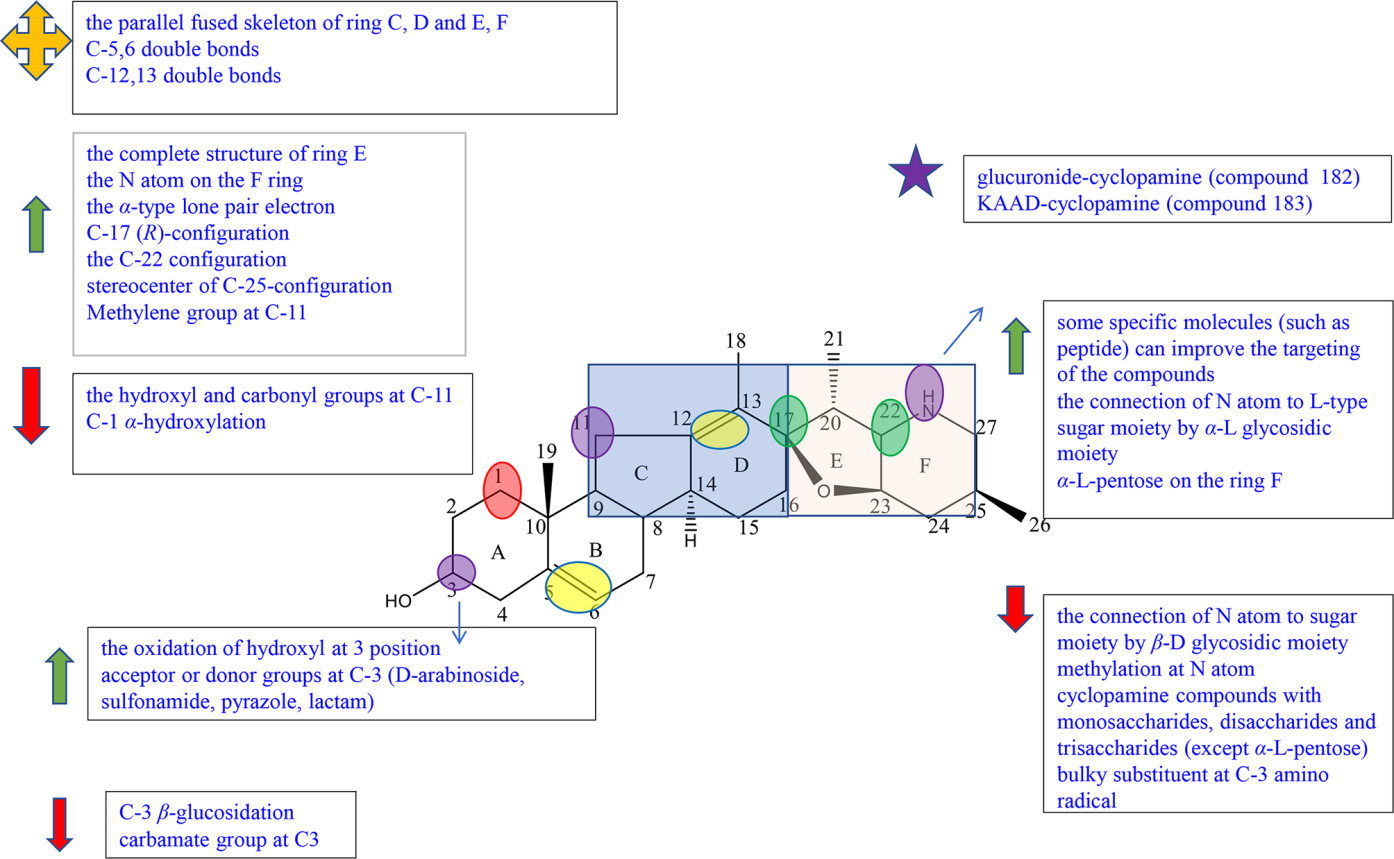
Summary of Structure-activity Relationship of Cyclopamine.

**Figure 7:**
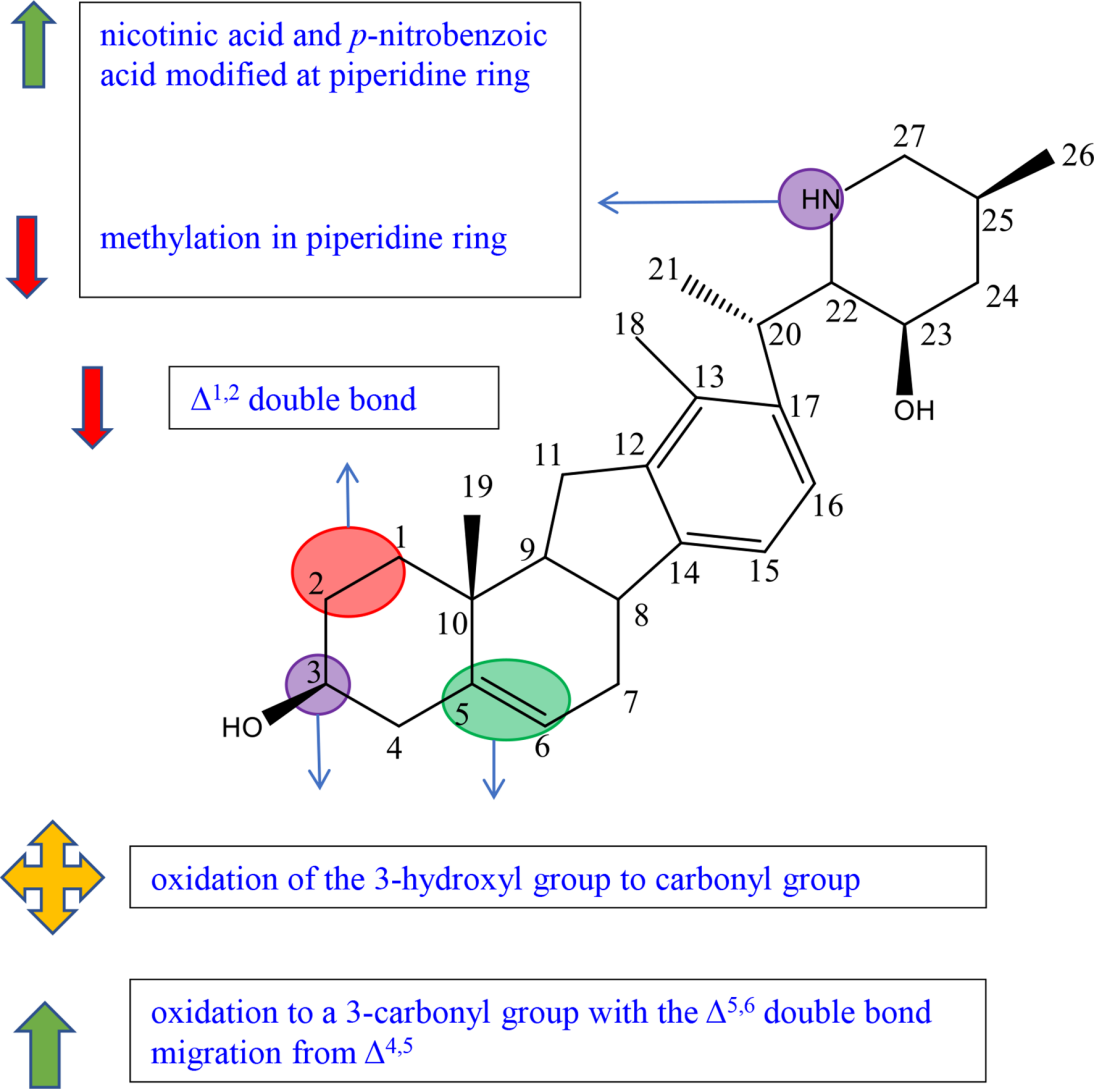
Summary of Structure-activity Relationship of Veratramine.

C-nor-D-Homosteroidal alkaloids are the other type of C-27 steroidal alkaloids. According to whether the E-ring is open or not, we divided C-nor-D-Homosteroidal alkaloids into cyclopamines and veratramines. The schematic diagram of SAR of cyclopamine is shown in **Figure 6** and that of veratramine is shown in **Figure 7**.

### Cyclopamine

Cyclopamine (**143**) is composed of four rings (named C-nor-D-homosteroid including rings A, B, C, and D) joined at the C-17 position by the cyclic tetrahydrofuran and piperidine systems (namely rings E and F) (**Figure S8**).

A SAR study showed that the parallel fused skeleton of rings C, D, E, and F played a little role in the activity of the compounds, while the complete structure of ring E, the N atom on the F ring, and the *α*-type lone pair electron were necessary for the activity (46, 47). And the C-17 (*R*)-configuration and the C-22 configuration also are essential elements because these can affect the orientation of piperidinic nitrogen in space (47).

Zhang et al. completed the synthesis of cyclopamine derivatives (144a-k, 145a-k) by the method of “Click” chemistry in order to preliminarily construct a library of carbohydrate–cyclopamine conjugates (**Figure S8**). Compound 145f (IC_50 =_ 33 *μ*M) with *α*-L rhamnose linked to an atom on the F ring of cyclopamine owns higher solubility and antitumor activity against lung cancer cell line A549 than cyclopamine (IC_50 =_ 49 *μ*M). However, the antitumor activity of cyclopamine connected with *β*-D sugar including monosaccharides, disaccharides, and trisaccharides was significantly decreased (145a, 145d, 145k) or even inactive (145b, 145g-j) (46, 48). It can be concluded that the connection of the N atom to L-type sugar moiety by *α*-L glycosidic moiety can maintain high activity and greatly improve solubility; *β*-D glycosidic moiety may lead the decrease in activities (46). In 2008, Zheng et al. synthesized the cyclopamine analogues including compounds 146–151 from jervine through the methods of reduction, oxidation, and methylation, and tested the activity against human pancreatic cancer Aspc-1 and human gastric cancer SGC-7901. Compound 146 showed prominent inhibition activity against Aspc-1 and SGC-7901 cells, while its methylation products, compound 147 and compound 150, showed no activity, suggesting that methylation at the N atom on the F ring reduced inhibition effects (**Figure S8**) (49). A previous study also proved that the connection of the N atom on ring F to some specific molecules (such as peptide) could improve the targeting of the compound (46).

Exo-cyclopamine (152) with an exo-methylene group at positions 13 and 14 was synthesized by Giannis et al. in 2011, which has good activity and stable acid (**Figure S8**) (50). Because of the advantages, in 2013, Giannis et al. synthesized compounds 153 and 154 for further study, which had the same skeleton as compound 152 except for the substituents at C-25 (**Figure S8**) (51). Compared with compound 143 (cyclopamine) and compound 152 with the C-25 (*R*)-configured methyl group, compound 153 with the C25 (s)-configured methyl group and compound 154 with the Δ^25,27^ exocyclic double bond showed better inhibition effects against SHh-LIGHT II cells through expressing the Gli1-dependent luciferase. These results suggested that the stereocenter of C-25 configuration affects the Hh inhibition (47).

Jervine (compound 155) is an 11-oxo derivative of cyclopamine (**Figure S8**). In Zheng’s experiment, among the three reduction products at C-11 of Jervine (compound 143, compound 156, and compound 157), only cyclopamine (compound 143) with the C-11 methylene group showed good inhibitory activity, while compound 156 and compound 157 with the C-11 hydroxy group showed comparably weak inhibitory activity to Jervine (**Figure S8**). The results showed that the substituents at C-11 were closely related to the antitumor activity. The methylene group at C-11 enhanced the antitumor activity, and the hydroxyl and carbonyl groups decreased the activity (49).

Sinha et al. have reported that the oxidization of the hydroxyl group to a carbonyl moiety at C-3 enhanced the activity (46). Also, in the experiment of Zheng, only compound 146 had relatively good inhibitory activity on Aspc-1 and SGC-7901 cells, whereas the oxidation products and methylation products of Jervine (compounds 146–151) show little activities (**Figure S8**). This result also indicated the conclusion that the oxidation of hydroxyl at the C-3 position may enhance the antitumor activity (49). Khanfar et al. were interested in jervine and veratrum alkaloids due to the reported possible inhibition of the HH signal. They tested the activity of jervine analogues (compounds 158–160) and veratrum alkaloids (155, 161–165) including natural alkaloids, biocatalytic alkaloids, and semisynthetic alkaloids and preliminarily summarized the structure–activity relationships (**Figure S8**). Compound **158** showed no inhibitory effects against PC-3 cells at 50 *μ*M concentration, while most compounds exhibited inhibition activity. The results indicated that C-3 *β*-glucosidation may reduce the antiproliferative activity (52). Among compounds 155 and 158–165, except compound 158 and compound 165, all compounds exhibited excellent antimigratory activity against PC-3 cells at 50 *μ*M concentration, suggesting that C-3 *β*-glucosidation (158) or the reduction of Δ^5,6^ double bond (165) played a negative role in antimigratory activity (52). Thorson et al. established a library of cyclopamine glycosides with the general skeleton of compound 166 to evaluate the effect of nonmetabolic sugars on cyclodopamine (**Figure S8**). In all derivatives, 166 showed better results (3.5–12 times) on the Hh inhibitory effect against lung cancer cell line NCI-H460 than that of cyclopamine (143). Among them, compound 166a with D-arabinoside showed the highest potency (GI_50_ = 6.4 ± 0.5 *μ*M) (53). Compared with compound 167 (EC_50_ = 0.3 ± 0.05 *μ*M), compound 168 with sulfonamide at C-3 (EC_50_ = 0.007 ± 0.002 *μ*M), compound 169 with pyrazole at C-3 (EC_50_ = 0.013 ± 0.008 *μ*M), and compound 170 with lactam at C-3 (EC_50_ = 0.025 ± 0.005 *μ*M) exhibited better efficacy against C3H10T1/2 cell line, suggesting that acceptor or donor groups at C-3 may play an essential role in activities (**Figure S8**) (54).

Gu et al. summarized a SAR exploration according to the collation and analysis of a large number of literatures, indicating that the double bonds at positions 5–6 and 12–13 are not necessary for the activity of the compound (46). However, in the experiment of Tremblay et al., the decreased inhibition activities of C3H10T1/2 cell differentiation of synthetic compound 171 may be associated with the removal of the C-3 hydroxyl group or the reduction of the Δ^5,6^ double bond (**Figure S8**) (54).

In the experiment of Khanfar et al., compound 165 showed great inhibitory effects against PC-3 cells at the 50 *μ*M dose, while compound 160 with the same skeleton except *α*-hydroxylation at C-1 exhibited no antiproliferative activity, implying that the decrease in inhibitory effects may be due to *α*-hydroxylation at C-1 (**Figure S8**) (52).

Many studies focused on the modification of cyclopamine (143) in order to improve the activities. However, according to the patent of Beachy et al., derivatives of 143 in acidic conditions showed lower biological activities and stabilities. For example, compound 172 with the carbamate group at C-3 and compound 173 with a bulky substituent at amino radical significantly reduced the inhibitory effect of differentiation in C3H10T1/2 cells (**Figure S8**) (55).

In the course of the research, scientists had also found some compounds with potential for development. Compound 145f with *α*-L-pentose on the ring F synthesized by Zhang et al. had obvious inhibitory activity against lung cancer A450 cells with IC_50_ of 33 *μ*M in the preliminary biological activity test. And 145f had higher inhibitory activity on the Hedgehog signal pathway than that of control (cyclopamine) (48). Compound 174 reported by Goff et al., a glucuronide-cyclopamine compound, significantly reduced the survival rate of malignant glioma U87 cell line with IC_50_ of 21 *μ*M, which had similar effect with cyclopamine in the control group (IC_50_ = 15.5 *μ*M) (**Figure S8**). Compared with cyclopamine, compound 174 showed obvious low toxicity and high effectiveness, having a significant research value *in vivo* activity for further study (56). KAAD-cyclopamine (compound 175) synthesized by Philipp et al. was a derivative substituted on the F ring of cyclopamine, showing higher aqueous solubility, more prominent activity, and lower toxicity (**Figure S8**) (57).

### Veratramine

Veratramine (161) has a similar chemical structure with cyclopamine, and the biggest difference is that ring E of veratramine is opened (**Figure S8**).

Guo et al. synthesized and tested five veratrylamine analogues (compounds 176–180) from veratrylamine (161) and preliminarily summarized the structure–activity relationships (**Figure S8**). Compound 176, considered as the oxidation product at C-3 of compound 161 (veratramine), showed comparable antiproliferative activities against SGC-7901 and ASPC-1 compared to compound 161. The result revealed that oxidation of the 3-hydroxyl group to the carbonyl group of compound 161 did not affect the activity (58). It should be pointed out that, in the experiment of Mohammed et al., among veratranes (155, 158-165), only compound 163 exhibited great inhibitory effects at 10 *μ*M, suggesting that oxidation to a C-3 group with the Δ^5,6^ double bond migration from Δ^4,5^ led to the enhancement of antiproliferative activities. The chemical modification may remedy the decrease in activity caused by opened ring E (**Figure S8**) (52).

In the experiment of Guo et al., compounds 178, 179, and 180 exhibited comparable or slightly higher activities than cyclopamine or veratramine (**Figure S8**). Moreover, compounds 178 and 179 had significantly higher activity than cyclopamine and veratramine against ASPC-1. The results suggested that the introduction of nicotinic acid and p-nitrobenzoic acid modified at piperidine ring enhanced the inhibitory effects (58).

Guo et al. also reported that the activity of compound 177 determined as the dimethylated product was weaker than that of compound 176 (**Figure S8**). It was speculated that the decrease in activity was because methylation in piperidine ring obscured the possible active groups such as -OH and-NH (58).

Veratrum alkaloids compound 164 reported by Mohammed et al. with Δ^1,2^ double bond displayed the highest antimigratory activity against the PC-3 cell line, while compound 163 exhibited only moderate activity (**Figure S8**). The results suggest that Δ^1,2^ double bond played a positive role in antimigratory activity (52).

## Bis-Steroidal Pyrazine Alkaloids

**Figure 8:**
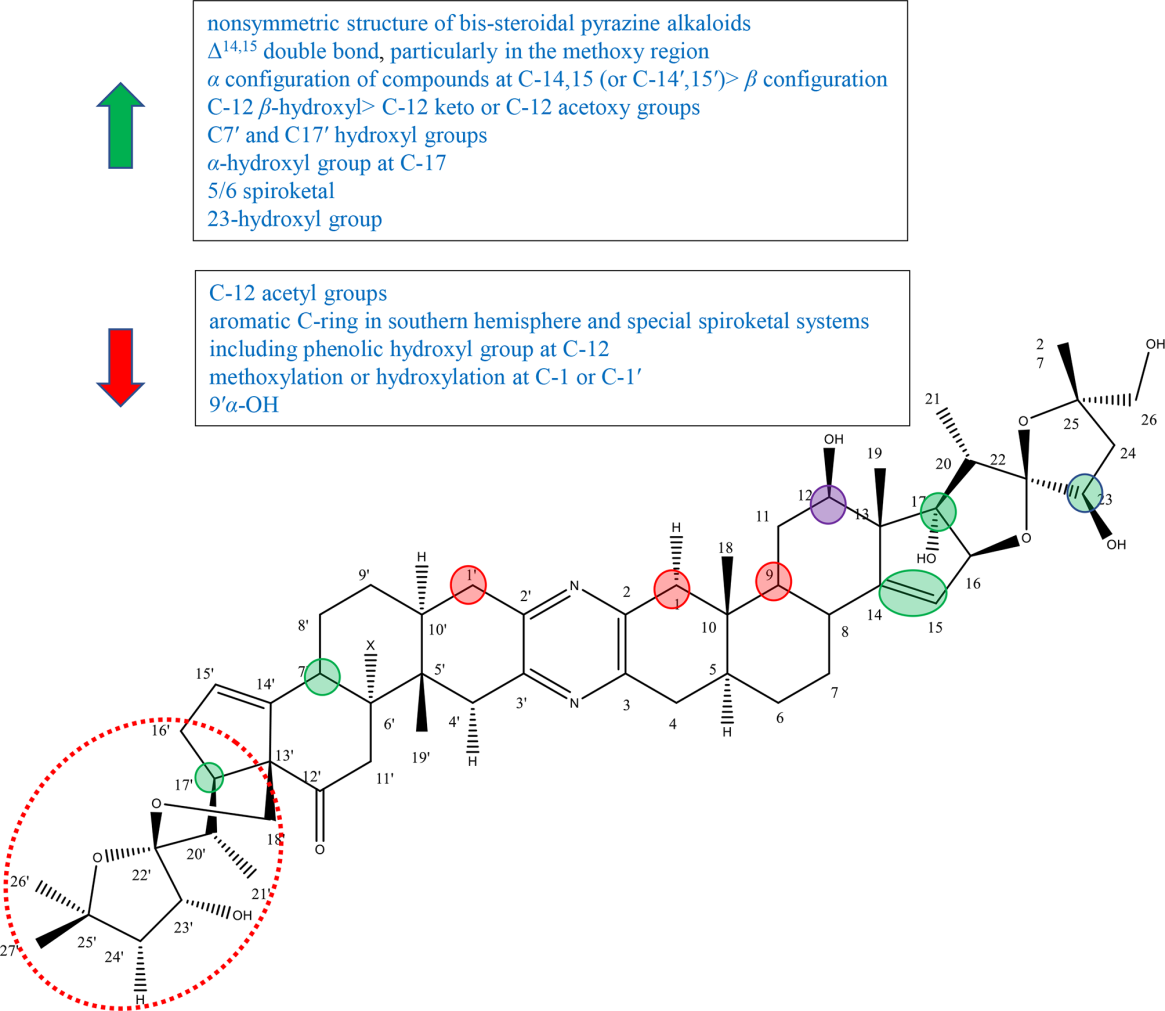
Summary of Structure-activity Relationship of Bis-steroidal Pyrazine Alkaloids.

Bis-steroidal pyrazine alkaloids, including cephalostatins isolated from marine tubeworm *Cephalodiscus gilchristi*, ritterazines isolated from marine organisms *Ritterella tokioka*, and their analogues, are a kind of steroid–alkaloid hybrids with a complex structure (59). The ritterazines and cephalostatins are dense ring compounds, which are fused by two steroidal spirocyclic units with 5/5 or 5/6 spiroketals *via* centra pyrazine heterocycles at C-2 and C-3 and show extremely significant antitumor activity (59–61). The schematic diagram of SAR of cyclopamine is shown in **Figure 8**.

The nonsymmetric structure of bis-steroidal pyrazine alkaloids played an important role in activities. In 2013, Iglesias-Arteaga et al. published a review on cephalostatins and ritterazines by summarizing the literature in 2012 and before. Iglesias-Arteaga et al. reported that symmetrical cephalostatins and ritterazines showed inferior activity, and unsymmetrical compounds were more active. For example, cephalostatin 12 (compound 192) and ritterazine K (compound 210) with polar units as well as ritterazines N (compound 213) and R (compound 217) with nonpolar units showed decreased anticancer activity, although they consist of the most powerful potent core units [including northern and southern hemispheres of cephalostatin 1, southern hemisphere of cephalostatin 7 (compound 187), and northern hemisphere of ritterazine G (compound 206)]. And cephalostatin 13 (compound 193), ritterazines J (compound 209), ritterazines L (compound 211), ritterazines M (compound 212), ritterazines O (compound 214), and ritterazines S (compound 218), which are nearly symmetric, exhibited lower activity (**Figure S9**). In addition, the most potent compounds contained significantly different steroidal portions (62).

Nawasreh continued to study the synthesis of multihydroxylated cephalostatin analogues and synthesized compound 226, compound 227, and borohydride compound 228 of compound 226. Compound 227 and compound 226 with Δ^14,15^ double bond showed relatively good activity against HM 02, HEP G2, and MCF 7, while compound 228 lacking it showed very weak activity (**Figure S9**) (63). In addition, in the review of Iglesias-Arteaga et al., most of bis-steroidal pyrazine alkaloids contained Δ^14,15^ double bond on at least one side, and most cephalostatins contained it on both sides (62). These results suggested that Δ^14,15^ double bond was important for biological activity, particularly in the methoxy region (62–64). Ritterazine I (compound 208) with 14*β*-H and 14*β*-OH was more active than ritterazine Z (compound 225) with 14′*α*-H or ritterazine U (compound 220) with 14′*α*-OH. As well as cephalostatin 4 (compound 184) with 14′*β*,15′*β*-epoxide was more active than cephalostatins 14 (compound 194) or cephalostatins 15 (compound 195) with 14′*α*,15′*α*-epoxides (**Figure S9**). The results suggested that the *α* configuration of the compounds at C-14,15 (or C-14′,15′) was superior to *β* configuration (62).

There are five common motifs (I–V) that presented most of alkaloids and three rare motifs (VI–VIII) (**Figure S10**) by Iglesias-Arteaga et al. Compounds with *β*-hydroxyl or carbonyl groups at 12 and 12′ positions are active. And the hydroxyl group appears to be the intrinsic group of units I, III, and IV, while the ketone group is the intrinsic functional group of units II and V (62). In 1997, Fukuzawa et al. isolated ritterazines N-Z (compounds 212–225) from *Ritterella tokioka* and modified these by reduction, methanolysis, oxidation, and acetylation to obtain compounds 229–236 (**Figure S9**). Ritterazine H (compound 207), determined as the oxidation derivative at C-12 of ritterazine B (compound 201), showed inferior activity to ritterazine B (compound 201), suggesting the importance of the C-12 hydroxyl group (64). LaCour et al. had reported that C-12 *β*-hydroxyl in North G played a positive role in the activity, since compounds with C-12 *β*-hydroxy had invariably higher activity than their counterparts with C-12 keto or C-12 acetoxy groups (65). By comparing with compounds 232–236, Fukuzawa et al. found that the more acetyl groups to introduce, the weaker the activity to exhibit, further proving the importance of the hydroxyl group at C-12 (64). However, in the review of Iglesias-Arteaga et al., compounds containing the aromatic C-ring in the southern hemisphere and special spiroketal systems including the phenolic hydroxyl group at C-12 showed inferior activity, such as cephalostatins 5 (compound 185) and 6 (compound 186) (62).

In the experiment of Fukuzawa et al., ritterazine Y (compound 224) and ritterazine B (compound 201) had the same skeleton except substituent groups at C7′ and C17′. Ritterazine B (compound 201) containing hydroxyl groups at C7′ and C17 was more active than ritterazine B (compound 201) without hydroxyl groups, suggesting that C7′ and C17′ hydroxyl groups may play a positive role in the activity (64). However, Iglesias-Arteaga et al. showed that additional hydroxylation at C-7′ or C-9′ played no or little role in the activity, while it slightly reduced the activity in some situations (9′*α*-OH) (62). Therefore, it was speculated that the C-17 hydroxyl group plays an important role in the activity and the C7′ hydroxyl group has a little effect.

It was mentioned in Iglesias-Arteaga ‘s article that the *α*-hydroxyl group at C-17 was the intrinsic group of core unit I and is often present in unit III (polar domains), but not in units II, IV, and V (nonpolar domains). The active alkaloids contain at least one *α*-hydroxyl group at C-17 or C-17′ (62). Gryszkiewicz-Wojtkielewicz et al. also reported that the *α*-hydroxyl group was considered to be a potential functional group (61). By comparing ritterazines T (compound 219) with ritterazines A (compound 200), and ritterazines Y (compound 224) with ritterazines B (compound 201), it can be concluded that the activity of compounds with the 17*α*-OH group (or 17′*α*-OH group) was significantly superior to that of analogous compounds without this group (62). In 1998, LaCour et al. synthesized ritterostatin G_N_1_N_ (237), ritterostatin G_N_1_S_ (238), and cephalostatin 1(181) totally for the first time (**Figure S9**). Ritterostatin G_N_1_S_ lacking the 17*α*-hydroxyl group was far less active than ritterostatin G_N_1_N_, which further verified the importance of the 17*α*-hydroxyl group (65).

Fukuzawa et al. reported that 5/6 spiroketal markedly contributed to cytotoxic activity through comparing the cytotoxicity of ritterazines B (compound 201) and C (compound 202). And the cytotoxicity of compounds 229, 230, and 231 also supported this conclusion (**Figure S9**). Therefore, it was speculated that the arrangement of 5/6 spiroketal was important for cytotoxicity (64).

Kumar et al. synthesized 23-deoxy-25-epi ritterostatin G_N_1_N_ (compound 239) and tested the activities against NCI-60 cancer cell lines. The results showed that 25-epi ritterostatin G_N_1_N_ (239) was 50- to 1,000-fold more effective in bioactivity evaluation than that of 23-deoxy-25-epi ritterostatin G_N_1_N_ (compound 240), which suggested that the 23-hydroxyl group of cephalostatin and ritterazine played an important role (**Figure S9**) (61). Iglesias-Arteaga et al. summed up the conclusion through cephalostatins 1 (181), 10 (190), 11 (191), 13 (193), 18 (198), and 19 (199) that methoxylation or hydroxylation at C-1 or C-1′ led to a decrease in the activity (**Figure S9**) (62).

## Conclusion

Steroidal alkaloids have good anticancer potential. However, due to the imperfect research and its own side effects, the application of steroidal alkaloids is limited. Therefore, there is an urgent need to force us to study the extraction, separation, structural modification, and pharmacological effects of steroidal alkaloids.

To aid the discovery of steroidal alkaloids, we summarize the steroid alkaloids with anticancer activity and conclude the structure–activity relationships. According to the preliminary SAR results, the cytotoxic activity of pregnane alkaloids is affected by C-6, C-7, C-5, C-6, C-3, C-7, C-15, and C-21 substituents. The C-3 position was the most important site to affect the activity; the substituent and configuration of C-3 are both important influential factors. Many scientists had focused on the modification of C-3. According to their results, 10f (IC_50_ value = 0.03 *μ*M) is the most potential compound to treat cancer. Meanwhile, C-16 and C-17 are important active sites. It can be concluded that the double bond at C-16, 17, the epoxidation at C-16, 17, and the hydroxyl group at C-16 can all improve anticancer activities. Besides, the double bond at C-5 and C-6 also can improve anticancer activities, and the substituent groups at C-21 are also key factors for cytostatic efficacy.

The main sources of cyclopregnane alkaloids are cortistatins from marine sponge *Corticium* simplex and cyclopregnane alkaloids from the family Buxus. Research on the anticancer activity of cyclopregnane alkaloids is more focused on cortistatins, but there are a few reports on cyclopregnane alkaloids from the family Buxus. Important factors affecting the activity of cyclopregnane alkaloids include isoquinoline moiety, the plane construction of the tetracyclic core part, and the resemblance in structure compared to cortistatin A.

In terms of structure, cholestane alkaloids are the most variable alkaloids in terms of structure among steroidal alkaloids, which can be divided into solasodine and other types. Both the aglycone and sugar residues are critical for cytotoxic activities. The factors related to sugar residues affect the activity of solasodine including the number of sugars, the number of *α*-L-rhamnose, the position of *α*-L-rhamnose, the order of sugar, and the type of sugar. The C-6 position of cholestane is the focus during the research. Scientists changed the substituents at C-6 to compare their activities. Dendrogenin A (DDA) containing imidazol joined with 6-ethylamino is a potential derivative.

For C-nor-D-Homosteroidal alkaloids, people pay more attention to cyclopamine. The high activity of cyclopamine is affected by many factors, including the complete structure of ring E, the N atom on the F ring, the *α*-type lone pair electron, C17 (*R*)-configuration, the C-22 configuration, and the stereocenter of C25-configuration. The ways to improve the activity of cyclopamine include methylation at C-11, the oxidation of hydroxyl at C-3, and the presence of acceptor or donor groups at C-3. However, cyclopamine is limited due to its high toxicity. KAAD-cyclopamine is a potential analogue of cyclopamine, showing higher aqueous solubility, more prominent activity, and lower toxicity.

Scientists’ research on bis-steroidal pyrazine alkaloids is focused on cephalostatins and ritterazines. According to the preliminary SAR, the ways to improve biological activity are the nonsymmetric structure of bis-steroidal pyrazine alkaloids and the presence of these groups including Δ14,15 double bond, *α* configuration of compounds at C-14,15 (or C-14′,15′), C-12 *β*-hydroxyl, C7′ and C17′ hydroxyl groups, *α*-hydroxyl group at C-17, 5/6 spiroketal, and the 23-hydroxyl group.

The review shows a summary concerning extensive SAR of steroidal alkaloids including pregnane alkaloids, cyclopregnane alkaloids, cholestane alkaloids, C-nor-D-homosteroidal alkaloids, and bis-steroidal pyrazine in the area of anticancer activity. This review is convenient for more scientists to modify steroidal alkaloids and the analogues, as well as to conduct more *in vivo* and preclinical studies to achieve the purpose of reducing toxicity and increasing efficiency. And it greatly saves time to find more active and selective drugs and makes it possible to design new and effective anticancer drugs in the short term.

## Author Contributions

YZ and HY conceived the project. YH reviewed the literature and drafted the article. GL contributed to classifying the literature. CH and XZ revised the manuscript. All authors contributed to the article and approved the submitted version.

## Funding

This work was supported by the National Natural Science Foundation of China [No. 81873089] and Scientific Research Projects of Tianjin Education Commission [No. 2018KJ004], the National Key Research and Development Program of China [2021YFE0203100] and the Natural Science Foundation of Liaoning [2021-MS-214]. The funders had no role in the study design, data collection and analysis, decision to publish, or preparation of the manuscript.

## Conflict of Interest

The authors declare that the research was conducted in the absence of any commercial or financial relationships that could be construed as a potential conflict of interest.

## Publisher’s Note

All claims expressed in this article are solely those of the authors and do not necessarily represent those of their affiliated organizations, or those of the publisher, the editors and the reviewers. Any product that may be evaluated in this article, or claim that may be made by its manufacturer, is not guaranteed or endorsed by the publisher.
