# Supplementary material for: Potential of Steroidal Alkaloids in Cancer: Perspective Insight Into Structure–Activity Relationships
Source: Front Oncol. 2021 Sep 20;11:733369. doi: 10.3389/fonc.2021.733369 (PMC8489381; doi:10.3389/fonc.2021.733369)
Supplement: Supplementary file 1 [file DataSheet_1.docx]

**Supporting Information**

**List of supplementary content**

**Figure S1**. the Structure of Pregnane Alkaloids

**Figure S2.** the Structure of Cyclopregnane Alkaloids in *Corticium*

**Figure S3**. the Structure of Analogues of Cortistatin A

**Figure S4**. the Structure of Cyclopregnane Alkaloids in Genus *Buxus*

**Figure S5**. the Structure of Cholestane Alkaloid—Solasodine (glycoalkaloid)

**Figure S6**. the Structure of Cholestane Alkaloid—Solasodine (not glycoalkaloid)

**Figure S7**. the Structure of Cholestane Alkaloid (Others)

**Figure S8**. the Structure of C-nor-D-Homosteroidal Alkaloids

**Figure S9**. the Structure of Bis-steroidal pyrazine alkaloids

**Figure S10**. the Structure of motifs of Bis-steroidal Pyrazine Alkaloids

**Table S1**. The biological activity evaluation of pregnane alkaloids against cancer cells.

**Table S2**. The biological activity evaluation of cyclopregnane alkaloids against cancer cells.

**Table S3**. The biological activity evaluation of solasodine (glycoalkaloid) against cancer cells.

**Table S4**. The biological activity evaluation of solasodine (not glycoalkaloid) against cancer cells.

**Table S5**. The biological activity evaluation of cholestane alkaloids (others) against cancer cells.

**Table S6**. The biological activity evaluation of C-nor-D-Homosteroidal alkaloids against cancer cells.

**Table S7**. The biological activity evaluation of bis-steroidal pyrazine alkaloids against cancer cells.

**Figure S1. the Structure of Pregnane Alkaloids**

**Figure S2. the Structure of Cyclopregnane Alkaloids in *Corticium***

**Figure S3. the Structure of Analogues of Cortistatin A**

**Figure S4.** **the Structure of Cyclopregnane Alkaloids in Genus *Buxus***

**Figure S5. the Structure of Cholestane Alkaloid—Solasodine (glycoalkaloid)**

**Figure S6.** **the Structure of Cholestane Alkaloid—Solasodine (not glycoalkaloid)**

**Figure S7.** **the Structure of Cholestane Alkaloid (Others)**

**Figure S8.** **the Structure of C-nor-D-Homosteroidal Alkaloids**

**Figure S9. the Structure of Bis-steroidal Pyrazine Alkaloids**

**Figure S10. the Structure of motifs of Bis-steroidal Pyrazine Alkaloids**

**Table S1**

**The biological activity evaluation of pregnane alkaloids against cancer cells.**

| Steroidal alkaloids | Cancer cell lines studied | IC_50_ (*μ*M) | References |
| --- | --- | --- | --- |
| **1** | SW480 | 10.97 ± 1.36 | 12 |
| **1** | SMMC-7721 | 41.31 ± 3.02 | 12 |
| **1** | PC3 | 32.97 ± 3.78 | 12 |
| **1** | K562 | 11.86 ± 0.82 | 12 |
| **2** | SW480 | 5.97 ± 0.13 | 12 |
| **2** | SMMC-7721 | 16.19 ± 0.56 | 12 |
| **2** | PC3 | 11.57 ± 0.86 | 12 |
| **2** | K562 | 7.95 ± 0.02 | 12 |
| **3** | SW480 | 5.77 ± 0.29 | 12 |
| **3** | SMMC-7721 | 10.84 ± 1.19 | 12 |
| **3** | PC3 | 11.79 ± 2.96 | 12 |
| **3** | K562 | 6.29 ± 0.53 | 12 |
| **4** | SW480 | 45.92 ± 1.56 | 12 |
| **4** | SMMC-7721 | 71.13 ± 5.37 | 12 |
| **4** | PC3 | >100 | 12 |
| **4** | K562 | 85.48 ± 6.77 | 12 |
| **5a** | MDA-MB-231 | 0.09 | 13 |
| **6a** | MDA-MB-231 | 2.21 | 13 |
| **5b** | MDA-MB-231 | 1.21 | 13 |
| **6b** | MDA-MB-231 | >50 | 13 |
| **5c** | MDA-MB-231 | 0.17 | 13 |
| **6c** | MDA-MB-231 | 20.15 | 13 |
| **5d** | MDA-MB-231 | 5.51 | 13 |
| **6d** | MDA-MB-231 | 7.45 | 13 |
| **5e** | MDA-MB-231 | 4.36 | 13 |
| **6e** | MDA-MB-231 | 24.23 | 13 |
| **5f** | MDA-MB-231 | >50 | 13 |
| **6f** | MDA-MB-231 | 2.48 | 13 |
| **5g** | MDA-MB-231 | 26.74 | 13 |
| **6g** | MDA-MB-231 | >50 | 13 |
| **7a** | MDA-MB-231 | Tox | 13 |
| **8a** | MDA-MB-231 | 1.08 | 13 |
| **7b** | MDA-MB-231 | 4.77 | 13 |
| **8b** | MDA-MB-231 | Tox | 13 |
| **7c** | MDA-MB-231 | Tox | 13 |
| **8c** | MDA-MB-231 | 32.84 | 13 |
| **7d** | MDA-MB-231 | Tox | 13 |
| **8d** | MDA-MB-231 | >50 | 13 |
| **7e** | MDA-MB-231 | Tox | 13 |
| **8e** | MDA-MB-231 | 0.26 | 13 |
| **7f** | MDA-MB-231 | 21.39 | 13 |
| **8f** | MDA-MB-231 | 0.85 | 13 |
| **7g** | MDA-MB-231 | 15.92 | 13 |
| **8g** | MDA-MB-231 | Tox | 13 |
| **7a** | MDA-MB-231 | 0.48 | 13 |
| **8a** | MDA-MB-231 | 0.30 | 13 |
| **9b** | MDA-MB-231 | 18.93 | 13 |
| **10b** | MDA-MB-231 | Tox | 13 |
| **9c** | MDA-MB-231 | 28.54 | 13 |
| **10c** | MDA-MB-231 | Tox | 13 |
| **9d** | MDA-MB-231 | 2.14 | 13 |
| **10d** | MDA-MB-231 | 7.37 | 13 |
| **9e** | MDA-MB-231 | Tox | 13 |
| **10e** | MDA-MB-231 | >50 | 13 |
| **9f** | MDA-MB-231 | >50 | 13 |
| **10f** | MDA-MB-231 | 0.03 | 13 |
| **9g** | MDA-MB-231 | 0.84 | 13 |
| **10g** | MDA-MB-231 | 2.34 | 13 |
| **11** | HL-60 | 5.48 | 14 |
| **12** | HL-60 | 1.74 | 14 |
| **13** | HL-60 | 7.63 | 14 |
| **14** | HT-29 | 31.06 | 15 |
| **14** | HeLa | 51.42 | 15 |
| **14** | MCF-7 | 42.82 | 15 |
| **14** | KMST-6 | 102.95 | 15 |
| **15** | HT-29 | 22.36 | 15 |
| **15** | HeLa | 46.17 | 15 |
| **15** | MCF-7 | 52.69 | 15 |
| **15** | KMST-6 | 85.45 | 15 |
| **16** | KB cells | 12.8 | 16 |
| **17** | KB cells | >50 | 16 |
| **18** | KB cells | 21-42 | 16 |
| **19** | KB cells | 21-42 | 16 |
| **20** | KB cells | >50 | 16 |
| **21** | NCI-H187 | 18.2 | 17 |
| **22** | NCI-H187 | Inactive | 17 |
| **23** | HL-60 | 0.2(*µ*g/mL) | 18 |
| **23** | P-388 | 0.91(*µ*g/mL) | 18 |
| **23** | P388/ADR | 0.85(*µ*g/mL) | 18 |
| **23** | P-388/VCR | 0.83(*µ*g/mL) | 18 |
| **24** | HL-60 | 1.2(*µ*g/mL) | 18 |
| **24** | P-388 | 4.1(*µ*g/mL) | 18 |
| **24** | P388/ADR | 3.6(*µ*g/mL) | 18 |
| **24** | P-388/VCR | 1.4(*µ*g/mL) | 18 |

**Table S2**

**The biological activity evaluation of cyclopregnane alkaloids against cancer cells.**

| Steroidal alkaloids | Cancer cell lines studied | IC_50_(*μ*M) | References |
| --- | --- | --- | --- |
| **25** | HUVEC | 0.0018 | 19-22 |
| **25** | KB3-1 | 7.0 | 19-22 |
| **25** | K562 | 6.0 | 19-22 |
| **25** | Neuro2A | 7.0 | 19-22 |
| **25** | NHDF | 6.0 | 19-22 |
| **26** | HUVEC | 1.1 | 19 |
| **26** | KB3-1 | 120 | 19 |
| **26** | K562 | 160 | 19 |
| **26** | Neuro2A | 200 | 19 |
| **26** | NHDF | >300 | 19 |
| **27** | HUVEC | 0.019 | 19 |
| **27** | KB3-1 | 150 | 19 |
| **27** | K562 | 180 | 19 |
| **27** | Neuro2A | >300 | 19 |
| **27** | NHDF | >300 | 19 |
| **28** | HUVEC | 0.15 | 19 |
| **28** | KB3-1 | 55 | 19 |
| **28** | K562 | >300 | 19 |
| **28** | Neuro2A | >300 | 19 |
| **28** | NHDF | >300 | 19 |
| **29** | HUVEC | 0.45 | 19 |
| **29** | KB3-1 | 2.5 | 19 |
| **29** | K562 | 1.9 | 19 |
| **29** | Neuro2A | 2.8 | 19 |
| **29** | NHDF | 1.9 | 19 |
| **30** | HUVEC | 1.9 | 19 |
| **30** | KB3-1 | 10.8 | 19 |
| **30** | K562 | 4.0 | 19 |
| **30** | Neuro2A | 4.0 | 19 |
| **30** | NHDF | 4.1 | 19 |
| **31** | HUVEC | 0.8 | 19 |
| **31** | KB3-1 | 8.9 | 19 |
| **31** | K562 | 4.0 | 19 |
| **31** | Neuro2A | 3.8 | 19 |
| **31** | NHDF | 2.9 | 19 |
| **32** | HUVEC | 0.35 | 19 |
| **32** | KB3-1 | 2.3 | 19 |
| **32** | K562 | 2.2 | 19 |
| **32** | Neuro2A | 2.7 | 19 |
| **32** | NHDF | 2.7 |  |
| **33** | HUVEC | 0.008 | 19 |
| **33** | KB3-1 | 9.1 | 19 |
| **33** | K562 | 3.3 | 19 |
| **33** | Neuro2A | 3.3 | 19 |
| **33** | NHDF | 2.4 | 19 |
| **34** | HUVEC | 0.04 | 19 |
| **34** | KB3-1 | 10.2 | 19 |
| **34** | K562 | 3.0 | 19 |
| **34** | Neuro2A | 3.9 | 19 |
| **34** | NHDF | 2.5 | 19 |
| **35** | HUVEC | 0.023 | 19 |
| **35** | KB3-1 | 14 | 19 |
| **35** | K562 | 2.8 | 19 |
| **35** | Neuro2A | 4.3 | 19 |
| **35** | NHDF | 2.4 | 19 |
| **38** | HUVEC | 2.0 | 20 |
| **38** | KB3-1 | 18 | 20 |
| **38** | K562 | NT | 20 |
| **38** | Neuro2A | NT | 20 |
| **38** | NHDF | NT | 20 |
| **39** | HUVEC | 0.1 | 20 |
| **39** | KB3-1 | 10.5 | 20 |
| **39** | K562 | 6 | 20 |
| **39** | Neuro2A | 10.5 | 20 |
| **39** | NHDF | 10.5 | 20 |
| **40** | HUVEC | 15 | 20 |
| **40** | KB3-1 | 20 | 20 |
| **40** | K562 | NT | 20 |
| **40** | Neuro2A | NT | 20 |
| **40** | NHDF | NT | 20 |
| **41** | HUVEC | 0.035 | 20 |
| **41** | KB3-1 | 10.5 | 20 |
| **41** | K562 | 5.0 | 20 |
| **41** | Neuro2A | 10.5 | 20 |
| **41** | NHDF | 4.0 | 20 |
| **36** | HUVEC | 0.035 | 22 |
| **36** | KB3-1 | 10.5 | 22 |
| **37** | HUVEC | 1.5 | 22 |
| **37** | KB3-1 | 19.2 | 22 |
| **42** | K562 | 0.37 | 23 |
| **43** | K562 | 4.44 | 23 |
| **44** | K562 | 1.70 | 23 |
| **45** | K562 | 5.61 | 23 |
| **46** | K562 | 2.95 | 23 |
| **47** | HL-60/SMMC-7721/A549/MCF-7/SW480 | >40 | 23 |
| **48** | HL-60/SMMC-7721/A549/MCF-7/SW480 | >40 | 23 |
| **49** | HL-60/SMMC-7721/A549/MCF-7/SW480 | >40 | 23 |
| **50** | HL-60/SMMC-7721/A549/MCF-7/SW480 | >40 | 23 |

**Table S3**

**The biological activity evaluation of solasodine (glycoalkaloid) against cancer cells.**

| Steroidal alkaloids | Cancer cell lines studied | IC_50_(*μ*M) | References |
| --- | --- | --- | --- |
| **53** | MGC-803 | 25.2 | 24 |
| **54** | MGC-803 | 26.5 | 24 |
| **55** | MGC-803 | 8.77 | 24 |
| **56** | MGC-803 | inactive | 24 |
| **57** | MGC-803 | inactive | 24 |
| **58** | MGC-803 | 20.1 | 24 |
| **59** | VERO P35 | 24.03 | 25、26 |
| **59** | NIH3T3 | 22.33 | 25、26 |
| **59** | AGS | 11.2 | 25、26 |
| **59** | HT-29 | 10.83 | 25、26 |
| **59** | MCF-7 | 2.62 | 25、26 |
| **59** | MDA-MB-231 | 13.72 | 25、26 |
| **60** | VERO P35 | 53.94 | 25、26 |
| **60** | NIH3T3 | 47.6 | 25、26 |
| **60** | AGS | 21.19 | 25、26 |
| **60** | HT-29 | 16.36 | 25、26 |
| **60** | MCF-7 | 5.01 | 25、26 |
| **60** | MDA-MB-231 | 20.68 | 25、26 |
| **61** | MCF-7 | 8.2 | 25、26 |
| **62** | MCF-7 | 19.89 | 25、26 |
| **63** | HL-60 | >50 | 27 |
| **63** | U937 | >50 | 27 |
| **63** | Jurkat | >50 | 27 |
| **63** | K562 | >50 | 27 |
| **63** | HepG2 | >50 | 27 |
| **64** | HL-60 | >50 | 27 |
| **64** | U937 | >50 | 27 |
| **64** | Jurkat | >50 | 27 |
| **64** | K562 | >50 | 27 |
| **64** | HepG2 | >50 | 27 |
| **65** | HL-60 | >50 | 27 |
| **65** | U937 | >50 | 27 |
| **65** | Jurkat | >50 | 27 |
| **65** | K562 | >50 | 27 |
| **65** | HepG2 | >50 | 27 |
| **66** | HL-60 | 3.53 ± 0.37 | 27 |
| **66** | U937 | 9.31 ± 0.22 | 27 |
| **66** | Jurkat | 2.72 ± 0.44 | 27 |
| **66** | K562 | 8.75 ± 1.91 | 27 |
| **66** | HepG2 | 5.36 ± 0.64 | 27 |
| **67** | HL-60 | 33.32 ± 1.33 | 27 |
| **67** | U937 | 39.16 ± 1.61 | 27 |
| **67** | Jurkat | 12.85 ± 2.45 | 27 |
| **67** | K562 | 26.83 ± 2.85 | 27 |
| **67** | HepG2 | 17.33 ± 1.28 | 27 |
| **68** | Ehrlich | 74.20 ± 6.26 | 28 |
| **68a** | Ehrlich | inactive | 28 |
| **70** | PC3 | not active at 10*μ*M (inhibitory) | 30 |
| **70** | Hela | not active at 10*μ*M (inhibitory) | 30 |
| **70** | HepG2 | not active at 10*μ*M (inhibitory) | 30 |
| **71** | PC3 | not active at 10*μ*M (inhibitory) | 30 |
| **71** | Hela | not active at 10*μ*M (inhibitory) | 30 |
| **71** | HepG2 | not active at 10*μ*M (inhibitory) | 30 |
| **72** | PC3 | not active at 10*μ*M (inhibitory) | 30 |
| **72** | Hela | not active at 10*μ*M (inhibitory) | 30 |
| **72** | HepG2 | not active at 10*μ*M (inhibitory) | 30 |
| **73** | PC3 | 51.0% at 10*μ*M (inhibitory) | 30 |
| **73** | Hela | 54.3% at 10*μ*M (inhibitory) | 30 |
| **73** | HepG2 | 40.4% at 10*μ*M (inhibitory) | 30 |
| **74** | PC3 | not active at 10*μ*M (inhibitory) | 30 |
| **74** | Hela | not active at 10*μ*M (inhibitory) | 30 |
| **74** | HepG2 | not active at 10*μ*M (inhibitory) | 30 |
| **75** | PC3 | not active at 10*μ*M (inhibitory) | 30 |
| **75** | Hela | not active at 10*μ*M (inhibitory) | 30 |
| **75** | HepG2 | not active at 10*μ*M (inhibitory) | 30 |
| **76** | MGC803 | >50 | 31 |
| **76** | HepG2 | >50 | 31 |
| **76** | SW480 | >50 | 31 |
| **77** | MGC803 | >50 | 31 |
| **77** | HepG2 | >50 | 31 |
| **77** | SW480 | >50 | 31 |
| **78** | MGC803 | 17.69 ± 0.56 | 31 |
| **78** | HepG2 | 22.98 ± 0.91 | 31 |
| **78** | SW480 | 23.79 ± 1.42 | 31 |
| **79** | MGC803 | 7.02 ± 0.60 | 31 |
| **79** | HepG2 | 12.74 ± 0.70 | 31 |
| **79** | SW480 | 11.16 ± 0.28 | 31 |

**Table S4**

**The biological activity evaluation of solasodine (not glycoalkaloid) against cancer cells.**

| Steroidal alkaloids | Cancer cell lines studied | IC_50_(*μ*M) | References |
| --- | --- | --- | --- |
| **82** | PC3 | 18.3±3.2 | 32 |
| **83** | PC3 | >25 | 32 |
| **84** | PC3 | >20 | 32 |
| **85** | PC3 | >20 | 32 |
| **86** | PC3 | >20 | 32 |
| **87** | PC3 | ND | 32 |
| **88** | PC3 | >20 | 32 |
| **89** | PC3 | 10–20 | 32 |
| **90** | PC3 | >20 | 32 |
| **91** | PC3 | 3.91±0.87 | 32 |
| **92** | PC3 | >20 | 32 |
| **93** | PC3 | 10.8±2.1 | 33 |
| **94** | PC3 | >33.3 | 33 |
| **95** | PC3 | >33.3 | 33 |
| **96** | PC3 | >33.3 | 33 |
| **97** | PC3 | >33.3 | 33 |
| **98** | PC3 | >33.3 | 33 |
| **99** | PC3 | >20 | 33 |
| **100** | PC3 | ND | 33 |

**Table S5**

**The biological activity evaluation of cholestane alkaloids (others) against cancer cells.**

| Steroidal alkaloids | Cancer cell lines studied | IC_50_/GI_50_/LC_50_ (*μ*M) | References |
| --- | --- | --- | --- |
| **101** | NCI-60 | 11.5（GI_50_） | 34 |
| **102** | NCI-60 | 2.4（GI_50_） | 34 |
| **104** | NCI-60 | 1.4（GI_50_） | 34 |
| **105** | M-109 | 12.5*µ*g/mL | 35 |
| **106** | M-109 | >20*µ*g/mL | 35 |
| **107** | M-109 | 31.2*µ*g/mL | 35 |
| **108** | K562 | 0.9*µ*g/mL（LC_50_） | 36 |
| **109** | K562 | 0.2*µ*g/mL（LC_50_） | 36 |
| **110** | K562 | 1.3*µ*g/mL（LC_50_） | 36 |
| **111** | MCF7 | >100 | 37 |
| **111a** | MCF7 | 12 | 37 |
| **112** | MCF7 | >100 | 37 |
| **112a** | MCF7 | 3 | 37 |
| **113** | MCF7 | 3 | 37 |
| **114** | MCF7 | 20 | 37 |
| **103** | HCT-116 | 10.6 | 38 |
| **104** | HCT-116 | 6.1 | 38 |
| **115** | HCT-116 | 1.4 | 38 |
| **116** | HCT-116 | 1.4 | 38 |
| **117** | HeLa | 4.2 | 39 |
| **118** | HeLa | >10 | 39 |
| **119** | HeLa | 3.6 | 39 |
| **120** | HeLa | >10 | 39 |
| **121** | HeLa | 4.9 | 39 |
| **122** | HeLa | 4.7 | 39 |
| **122** | HEPG2 | 4.2 | 39 |
| **123** | HeLa | 2.2 | 39 |
| **123** | HEPG2 | 4.5 | 39 |
| **124** | HeLa | <10 | 39 |
| **125** | HeLa | <10 | 39 |
| **125** | HEPG2 | 8.4 | 39 |
| **126** | HeLa | >10 | 39 |
| **127** | HeLa | <10 | 39 |
| **128** | HeLa | <10 | 39 |
| **129** | HeLa | <10 | 39 |
| **130** | HeLa | <10 | 39 |
| **131** | MCF-7 | 2.31±0.2 | 40 |
| **131** | TSA^H2d^ | 1.28±0.2 | 40 |
| **131** | B16F10 | 0.68±0.1 | 40 |
| **131** | SK-MEL-28 | 2.12±0.2 | 40 |
| **131** | A549 | 2.56±0.6 | 40 |
| **131** | P19 | 1.80±0.4 | 40 |
| **131** | SK-N-SH | 2.24±0.5 | 40 |
| **131** | SH-SY5Y | 1.35±0.5 | 40 |
| **131** | Neuro2A | 2.32±0.2 | 40 |
| **131** | U87 | 0.74±0.2 | 40 |
| **131** | U937 | 0.79±0.3 | 40 |
| **131** | NB4 | 1.32±0.4 | 40 |
| **131** | KG1 | 5.53±0.8 | 40 |
| **131** | HCT-8 | 0.89±0.2 | 40 |
| **131** | SW620 | 0.42±0.1 | 40 |
| **131** | KGI | 5.5 | 41 |
| **132** | HL-60 | 10.02 | 42 |
| **132** | SMMC-7721 | 17.05 | 42 |
| **132** | A-549 | 16.51 | 42 |
| **132** | MCF-7 | 6.4 | 42 |
| **132** | SW480 | 5.61 | 42 |
| **133** | HL-60 | 10.69 | 42 |
| **133** | SMMC-7721 | >40 | 42 |
| **133** | A-549 | 19.08 | 42 |
| **133** | MCF-7 | 8.29 | 42 |
| **133** | SW480 | 11.53 | 42 |
| **134** | Shh-LIGHT 2 cells | 3.11±0.44 | 44 |
| **135** | Shh-LIGHT 2 cells | 2.78±0.21 | 44 |
| **136** | Shh-LIGHT 2 cells | 1.11±0.36 | 44 |
| **137** | Shh-LIGHT 2 cells | >25 | 44 |
| **138** | Shh-LIGHT 2 cells | 0.63 ± 0.02 | 44 |
| **139** | Shh-LIGHT 2 cells | >25 | 44 |
| **140** | Shh-LIGHT 2 cells | >25 | 44 |
| **141** | LLC | 3.03±0.32 | 45 |
| **141** | A2780 | 17.03±0.89 | 45 |
| **141** | HEPG2 | 14.64±0.65 | 45 |
| **141** | A549 | 47.39±1.90 | 45 |
| **142** | LLC | 66.26±3.46 | 45 |
| **142** | A2780 | 39.18±2.21 | 45 |
| **142** | HEPG2 | 84.26±4.46 | 45 |
| **142** | A549 | 117.84±6.29 | 45 |

**Table S6**

**The biological activity evaluation of C-nor-D-Homosteroidal alkaloids against cancer cells.**

| Steroidal alkaloids | Cancer cell lines studied | IC_50_/GI_50_/EC_50_ (*μ*M) | References |
| --- | --- | --- | --- |
| **143** | A549 | 49 | 46 |
| **146** | SGC- 7901 | 83.86 ±1.71 | 48 |
| **146** | ASPC-1 | 86.68 ±0.55 | 48 |
| **147** | SGC- 7901 | ＞100 | 48 |
| **147** | ASPC-1 | ＞100 | 48 |
| **148** | SGC- 7901 | ＞100 | 48 |
| **148** | ASPC-1 | ＞100 | 48 |
| **149** | SGC- 7901 | 78.20 ±0.62 | 48 |
| **149** | ASPC-1 | ＞100 | 48 |
| **150** | SGC- 7901 | ＞100 | 48 |
| **150** | ASPC-1 | ＞100 | 48 |
| **151** | SGC- 7901 | ＞100 | 48 |
| **151** | ASPC-1 | ＞100 | 48 |
| **152** | SHh-LIGHT II | 3.29±0.31 | 49 |
| **153** | SHh-LIGHT II | 0.20±0.01 | 49 |
| **154** | SHh-LIGHT II | 0.5 | 49 |
| **155** | SGC- 7901 | ＞100 | 48 |
| **155** | ASPC-1 | ＞100 | 48 |
| **143** | SGC- 7901（48h） | 52.47±0.69 | 48 |
| **143** | ASPC-1（48h） | 53.55±0.55 | 48 |
| **156** | SGC- 7901（48h） | ＞100 | 48 |
| **156** | ASPC-1（48h） | ＞100 | 48 |
| **157** | SGC- 7901（48h） | ＞100 | 48 |
| **157** | ASPC-1（48h） | ＞100 | 48 |
| **166a** | NCI-H460 | 6.4±0.5（GI_50_） | 53 |
| **167** | Osteoblast C3H10T1/2 | 0.3±0.05（EC_50_） | 54 |
| **168** | Osteoblast C3H10T1/2 | 0.007±0.002（EC_50_） | 54 |
| **169** | Osteoblast C3H10T1/2 | 0.013±0.008（EC_50_） | 54 |
| **170** | Osteoblast C3H10T1/2 | 0.025±0.005（EC_50_） | 54 |
| **171** | C3H10T1/2 | - | 55 |
| **172** | C3H10T1/2 | - | 56 |
| **173** | C3H10T1/2 | - | 56 |
| **174** | U87 | 21 | 57 |
| **143** | U87 | 15.5 | 57 |
| **175** | Fibroblasts SHh-LIGHT I | 0.050 | 58 |
| **161**) | SGC－7901 | 73.7±1.35 | 59 |
| **161** | ASPC－1 | 84.4±5.06 | 59 |
| **176** | SGC－7901 | 67.4±2.12 | 59 |
| **176** | ASPC—1 | 90.5±1.16 | 59 |
| **177** | SGC－7901 | ＞100 | 59 |
| **177** | ASPC－1 | ＞100 | 59 |
| **178** | SGC－7901 | 41.2±0.7 | 59 |
| **178** | ASPC－1 | 42.2±0.53 | 59 |
| **179** | SGC－7901 | 54.3 ±11.21 | 59 |
| **179** | ASPC－1 | 48.3 ±0.64 | 59 |
| **180** | SGC－7901 | 42.6 ±0.35 | 59 |
| **180** | ASPC－1 | 84.9 ±0.59 | 59 |

**Table S7**

**The biological activity evaluation of bis-steroidal pyrazine alkaloids against cancer cells.**

| Steroidal alkaloids | Cancer cell lines studied | IC_50_/GI_50_ (nM) | References |
| --- | --- | --- | --- |
| **181** | P388 | <0.0001 | 63 |
| **181** | NCI-60 | 1.2–4.2(GI_50_) | 63 |
| **182** | P388 | <0.0001 | 63 |
| **182** | NCI-60 | 0.78–6.5(GI_50_) | 63 |
| **183** | P388 | <0.0001 | 63 |
| **183** | NCI-60 | 4.0(GI_50_) | 63 |
| **184** | P388 | <0.0001 | 63 |
| **184** | NCI-60 | 36(GI_50_) | 63 |
| **185** | P388 | 4.2 | 63 |
| **185** | NCI-60 | 130(GI_50_) | 63 |
| **186** | P388 | 2.2 | 63 |
| **186** | NCI-60 | 320(GI_50_) | 63 |
| **187** | P388 | <0.0001 | 63 |
| **187** | NCI-60 | 6.5–76(GI_50_) | 63 |
| **188** | P388 | <0.0001 | 63 |
| **188** | NCI-60 | 9.7–29(GI_50_) | 63 |
| **189** | P388 | <0.0001 | 63 |
| **189** | NCI-60 | 6.3(GI_50_) | 63 |
| **190** | P388 | 3.2 | 63 |
| **190** | NCI-60 | 4.1(GI_50_) | 63 |
| **191** | P388 | 2.7 | 63 |
| **191** | NCI-60 | 11(GI_50_) | 63 |
| **192** | P388 | 76 | 63 |
| **192** | NCI-60 | 400(GI_50_) | 63 |
| **193** | P388 | 48 | 63 |
| **193** | NCI-60 | >1000(GI_50_) | 63 |
| **194** | P38 | 4.4 | 63 |
| **194** | NCI-60 | 100(GI_50_) | 63 |
| **195** | P388 | 27 | 63 |
| **195** | NCI-60 | 68(GI_50_) | 63 |
| **196** | P388 | <1 | 63 |
| **196** | NCI-60 | 1(GI_50_) | 63 |
| **197** | P388 | 4.6 | 63 |
| **197** | NCI-60 | 4(GI_50_) | 63 |
| **198** | P388 | 4.6 | 63 |
| **198** | NCI-60 | 22(GI_50_) | 63 |
| **199** | P388 | 7.9 | 63 |
| **199** | NCI-60 | 17(GI_50_) | 63 |
| **200** | P388 | 3.8 | 63 |
| **200** | NCI-60 | 24(GI_50_) | 63 |
| **201** | P388 | 0.17 | 63 |
| **201** | NCI-60 | 3.2(GI_50_) | 63 |
| **202** | P388 | 102 | 63 |
| **202** | NCI-60 | 115(GI_50_) | 63 |
| **203** | P388 | 18 | 63 |
| **203** | NCI-60 | 102(GI_50_) | 63 |
| **204** | P388 | 3.8 | 63 |
| **204** | NCI-60 | 37(GI_50_) | 63 |
| **205** | P388 | 0.81 | 63 |
| **205** | NCI-60 | —— | 63 |
| **206** | P388 | 0.81 | 63 |
| **206** | NCI-60 | —— | 63 |
| **207** | P388 | 18 | 63 |
| **207** | NCI-60 | —— | 63 |
| **208** | P388 | 15 | 63 |
| **208** | NCI-60 | 88(GI_50_) | 63 |
| **209** | P388 | 14 | 63 |
| **209** | NCI-60 | —— | 63 |
| **210** | P388 | 10 | 63 |
| **210** | NCI-60 | 70(GI_50_) | 63 |
| **211** | P388 | 11 | 63 |
| **211** | NCI-60 | 20(GI_50_) | 63 |
| **212** | P388 | 17 | 63 |
| **212** | NCI-60 | —— | 63 |
| **213** | P388 | 522 | 63 |
| **213** | NCI-60 | —— | 63 |
| **214** | P388 | 2380 | 63 |
| **214** | NCI-60 | Inactive | 63 |
| **215** | P388 | 819 | 63 |
| **215** | NCI-60 | —— | 63 |
| **216** | P388 | 657 | 63 |
| **216** | NCI-60 | —— | 63 |
| **217** | P388 | 2462 | 63 |
| **217** | NCI-60 | —— | 63 |
| **218** | P388 | 539 | 63 |
| **218** | NCI-60 | —— | 63 |
| **219** | P388 | 522 | 63 |
| **219** | NCI-60 | >590(GI_50_) | 63 |
| **220** | P388 | 2340 | 63 |
| **220** | NCI-60 | >243(GI_50_) | 63 |
| **221** | P388 | 513 | 63 |
| **221** | NCI-60 | —— | 63 |
| **222** | P388 | 3632 | 63 |
| **222** | NCI-60 | —— | 63 |
| **223** | P388 | 3405 | 63 |
| **223** | NCI-60 | —— | 63 |
| **224** | P388 | 4 | 63 |
| **224** | NCI-60 | 27(GI_50_) | 63 |
| **225** | P388 | 2200 | 63 |
| **225** | NCI-60 | 722(GI_50_) | 63 |
| **201** | P388 | 0.00015*µ*g/mL | 65 |
| **207** | P388 | 0.016*µ*g/mL | 65 |
| **224** | P388 | 0.0035*µ*g/mL | 65 |
| **229** | P388 | 2.1*µ*g/mL | 65 |
| **230** | P388 | 2.5*µ*g/mL | 65 |
| **231** | P388 | 0.24*µ*g/mL | 65 |
| **232** | P388 | 0.018*µ*g/mL | 65 |
| **233** | P388 | 0.8*µ*g/mL | 65 |
| **234** | P388 | 7.6*µ*g/mL | 65 |
| **235** | P388 | 0.09*µ*g/mL | 65 |
| **236** | P388 | 0.0035*µ*g/mL | 65 |
| **237** | NCI-60 | mean < -7.4 at 900 nM(GI_50_) | 66 |
| **238** | NCI-60 | mean > -6.1 at 900 nM(GI_50_) | 66 |
| **239** | Leukemia HL-60 | < 0.1 | 62 |
| **239** | Lung A-549 | < 0.1 | 62 |
| **239** | Colon HT-29 | 0.3 | 62 |
| **239** | CNS SF-295 | < 0.1 | 62 |
| **239** | Breast MCF-7 | < 0.1 | 62 |
| **239** | Ovarian SK-OV-3 | < 0.1 | 62 |
| **239** | Melanoma LOX IMVI | 0.7 | 62 |
| **239** | Renal A-498 | 0.2 | 62 |
| **239** | Prostate PC-3 | <0.1 | 62 |
| **240** | Leukemia HL-60 | 581 | 62 |
| **240** | Lung A-549 | 898 | 62 |
| **240** | Colon HT-29 | 359 | 62 |
| **240** | CNS SF-295 | 91.1 | 62 |
| **240** | Breast MCF-7 | 534 | 62 |
| **240** | Ovarian SK-OV-3 | 450 | 62 |
| **240** | Melanoma LOX IMVI | 647 | 62 |
| **240** | Renal A-498 | 252 | 62 |
| **240** | Prostate PC-3 | 434 | 62 |
